# Plant tissue type and mineral contents shape endophytic bacterial communities in the Sisrè berry plant [*Synsepalum dulcificum* (Schumach & Thonn.) Daniell] in Benin

**DOI:** 10.1371/journal.pone.0327715

**Published:** 2025-07-07

**Authors:** Rabiath F. R. Adigoun, Alexis Durand, Hervé N. S. Aholoukpè, Dèdéou A. Tchokponhoué, Enoch G. Achigan-Dako, Aimé H. Bokonon-Ganta, Emile Benizri

**Affiliations:** 1 Université de Lorraine, INRAE, LSE, Nancy, France; 2 Genetics, Biotechnology and Seed Science Unit (GBioS), Laboratory of Plant Production, Physiology and Plant Breeding (PAGEV), Department of Plant Sciences, Faculty of Agronomic Sciences, University of Abomey-Calavi, Abomey-Calavi, Republic of Benin; 3 Agricultural Entomology Unit (LEAg), Laboratory of Pest Management (LaGON), Department of Plant Sciences, Faculty of Agronomic Sciences, University of Abomey-Calavi, Abomey‑Calavi, Republic of Benin; 4 Centre de Recherches Agricoles Plantes Pérennes (CRA-PP), Institut National des Recherches Agricoles du Bénin, Pobè, Republic of Benin; University of Salento: Universita del Salento, ITALY

## Abstract

Diverse endophytic bacteria inhabit distinct tissues of a given species and are essential for plant growth and resilience to various stresses. Little information is available on bacterial endophytes associated with *Synsepalum dulcificum*, an opportunity fruit crop with high economic and medicinal values. Using Illumina sequencing of the bacterial 16S rRNA gene, the diversity and structure of the endophytic bacterial community in the roots and leaves of *S. dulcificum* were determined, considering 29 accessions from three distinct phenotypes located either in home gardens or on farms in Benin. 2,468 Operational Taxonomic Units (OTUs) were recorded in the leaf and root endosphere of *S. dulcificum*, affiliated with 20 bacterial phyla, 49 classes, 125 orders, 217 families and 365 genera. Actinomycetota, Pseudomonadota and Chloroflexota were the most abundant phyla in the roots. In comparison, Pseudomonadota stood out as almost the unique phylum in the leaves, suggesting a significant decrease in diversity from roots to leaves. Significant correlations (p < 0.05) were observed between the relative abundance of the endophytic bacterial taxa and the mineral contents in the leaves, roots, and soil. While bacterial communities depended highly on accession, plant phenotype and habitat discriminated them in roots and leaves, respectively. Metagenome function prediction indicated that *S. dulcificum* harbors bacteria with the potential to metabolize carbohydrates and amino acids, as well as synthesize secondary metabolites and antimicrobial compounds beneficial for plant growth and adaptation to environmental stresses. These findings open room for exploiting endophytic diversity to enhance the growth and sustainable production of *S. dulcificum*.

## Introduction

The Sisrè berry plant [*Synsepalum dulcificum* (Schumach. & Thonn.) Daniell] (Sapotaceae) is a global ever-rising attention horticultural plant due to its red fruits known to contain miraculin, a sweetening glycoprotein that has the property to change the sour taste to sweet [1]. The species has numerous applications in both modern medicine [e.g., cancer [2], diabetes [3], hyperlipidemia [4] and Alzheimer’s [5] diseases treatment] and traditional medicine [6]. It is also used in the cosmetics [7] and beverage industries [8]. These applications are attributable to its fruits, seeds, bark, and leaves, which contain several health-promoting compounds such as flavonoids, saponins, phenolic acids, lignans, triterpenoids, flavonoid glycosides, phytosterols and megastigmane glycosides [9–12]. Notably, the high economic value of *S. dulcificum* is highlighted by the cost of 1 kg of its dry pulp powder, which can reach up to USD 2,500 (https://www.miraclefruitfarm.com/supplements), demonstrating its potential for income generation.

*Synsepalum dulcificum* is an evergreen species and rarely exceeds 8 meters in height during its life cycle [13]. Native to West Africa, its center of diversity is located in the Dahomey Gap and the Upper Guinea regions of the West African rainforest [14]. It is currently found in home gardens, backyards, farm galleries, fallows and forests [15]. The species is slow growing [16], which affects the willingness of local communities as well as some investors to engage in its production. This is emphasized by the fact that its first flowers appear after four to five years under natural conditions [17], while early fruiting stood among the desired traits by farmers [15]. Using agronomic practices (water and inorganic fertilizer management), Tchokponhoué et al. [18] were able to improve the species growth and reduce the time to flowering from over 36 months to an average of 23 months, which is a significant achievement. A more sustainable way to improve the species’ growth could be through the use of its associated beneficial bacteria called plant growth-promoting bacteria (PGPB) [19]. Indeed, PGPB include plant growth-promoting rhizobacteria (PGPR) and plant growth-promoting endophytic bacteria (PGPE) [20]. While PGPR inhabit rhizosphere soil and plant root surfaces, PGPE colonize internal plant tissues, including leaves, stems, roots, seeds, pollens and fruits [21,22]. Endophytic bacteria establish specific symbiosis with host plants and may have an advantage over rhizosphere bacteria because they live inside plant tissues and interact directly and efficiently with their host, resulting in a direct beneficial effect [21]. Moreover, endophytic bacteria benefit from more stable environments as they are not subjected to changes in soil conditions like their rhizosphere counterparts. Like PGPR, and based on their functional properties, PGPE can affect plant growth directly or indirectly. Their direct effects on the host plants include the mobilization of nutrients (nitrogen, phosphorus and iron) from the environment and plant growth promotion through providing or regulating plant hormones (cytokinin, auxin or ethylene) [21,23]. In addition, PGPE may indirectly affect plant growth by mitigating plant pathogens’ damage through (i) synthesizing antibiotics, antifungal metabolites, extracellular enzymes and pathogen-inhibiting volatile compounds, (ii) decreasing the amount of iron available to pathogens and (iii) inducing systemic resistance [21,24]. Therefore, PGPE could play crucial role in ensuring agriculture sustainability, climate change mitigation and adaptation, as well as food security and nutrition. Furthermore, studies have demonstrated the efficacity of beneficial endophytic bacteria in the plant growth of horticultural crops such as mulberry tree (*Morus alba* L.) [25], cashew tree (*Anacardium othonianum* Rizzini) [26] and pineapple (*Ananas* spp) [27]. However, leveraging the potential of the plant-beneficial endophytic bacteria requires understanding and characterizing community diversity and composition in the targeted crop species. This is particularly crucial as each host plant evolves within a specific environment, with its associated microorganisms forming a unit of biological organization called the holobiont [28]. Furthermore, plant endophytic bacterial communities are influenced by many factors such as plant tissue, growth stage, nutritional status and health [29,30], as well as geographical locations, climatic conditions, agricultural practices and host genotype/cultivar [31–34].

Bacterial endophytes participate in regulating plant gene expression [35,36], plant hormone pathways [37], plant metabolite concentrations [38,39] and plant tissue nutrient concentrations [40]. The nutrient concentration of a plant’s roots and leaves is closely related to the plant’s growth, and both are affected by the soil’s fertility [41] Thus, understanding the link between the plant’s nutritional status and the composition of the endophytic bacterial communities is crucial to better understanding their potential to stimulate plant growth.

More recently, Liu et al. [42] described for the first time the diversity of uncultured and culturable endophytic bacteria and fungi associated with *S. dulcificum* in China. Their study demonstrated the potential of these endophytes to produce bioactive metabolites for their application in manufacturing antioxidants, antibiotics and hypoglycemic drugs. Although their research concluded on endophytes from roots, stems, leaves, flowers, and fruits, the samples used were collected from a single *S. dulcificum* plantation. Building on their findings and considering that different factors could affect endophytic microbial community diversity and composition, it would also be interesting to assess the diversity of *S. dulcificum* endophytic bacteria across different locations and habitats and from different accessions/genotypes in West Africa, where the species occurs naturally. This is particularly relevant since Tchokponhoué et al. [13] previously delineated at least three phenotypic groups in West Africa based on the tree- and fruit-traits variations in *S. dulcificum*. The first phenotypic group included individual trees with weak plant architecture and poor fruit quality. The second phenotypic group was characterized by individual trees with strong plant architecture and high seed mass. The third phenotypic group encompassed individual trees with weak tree architecture and high-performance fruit traits and considered as elite one [13]. Consequently, this study aimed first to characterize the diversity and composition of the endophytic bacterial community associated with *S. dulcificum* in Benin, second to unravel the relationship between *S. dulcificum* leaf and root mineral contents, soil chemical characteristics, and the endophytic bacterial community composition, and last to identify the potential functions of bacterial communities through metagenome function prediction.

## Materials and methods

### Study area and plant material sampling

The plant material consisted of healthy root and leaf samples collected in three replicates from 29 accessions (individual trees) of *S. dulcificum*, all located in the Guineo-congolian phytogeographical region of Benin Republic (S1 Table). In this study, an accession refers to a distinct and uniquely identifiable individual tree of *S. dulcificum* from which root and leaf samples were collected. These 29 accessions constituted a core collection developed from a whole set of 153 accessions previously described by Tchokponhoué et al. [13] in the Dahomey Gap for their phenotypic performance. The core set was built using the CoreHunter algorithm supported by the “average entry-to-nearest-entry distance” optimization strategy objective in the “CoreHunter3” R package [43]. The phenotypic characteristics of the 29 accessions of the core collection were as follows: 11 accessions with weak tree architecture and poor fruit quality (referring here as the phenotypic group 1), 9 accessions with strong tree architecture and high seed mass (phenotypic group 2) and 9 accessions considered as elite ones, with weak tree architecture and high-performance fruit traits (phenotypic group 3). The accessions were geographically distributed across the departments of Zou, Atlantique, Mono, Couffo, Ouémé and Plateau and recorded in two types of habitats: farms (31% of accessions) and home gardens (69% of accessions) (S1 Table). Root sampling involved collecting lateral and fine roots under the trees’ canopy over a 20 cm layer of soil after removing litter. Leaf sampling was conducted by collecting a mixture of both mature and juvenile leaves from each tree. A total of 87 root samples and 87 leaf samples were collected from the 29 accessions and immediately placed on ice, then transported to the laboratory and stored at 4 °C until further processing.

### Chemical analyses of plant material and soil chemical characteristics

Leaf and root subsamples (three subsamples per accession) were thoroughly washed three times with deionized water, dried at 40 °C for 48 h and ground into a powder. Then, 0.05 g of ground plant material was analyzed through digestion in 1 ml concentrated nitric acid (65%) and 2 ml hydrogen peroxide (30%) at 95 °C for two hours. The digest was diluted to 10 ml with distilled water for analysis with ICP-AES (Inductively Coupled Plasma-Atomic Emission Spectrometer; ICP-AES, Liberty II, Varian). Mineral elements included in the study were: phosphorus (P), potassium (K), calcium (Ca), magnesium (Mg), sulfur (S), sodium (Na), aluminum (Al), iron (Fe), manganese (Mn), zinc (Zn), arsenic (As), boron (B), cadmium (Cd), cobalt (Co), chromium (Cr), copper (Cu), nickel (Ni), and lead (Pb). Total carbon (C) and nitrogen (N) in the samples were quantified by combustion at 900 °C using a CHNS analyzer (vario MICRO cube, Elementar Analysensysteme GmbH). Furthermore, the data about the chemical characteristics [P, K, Ca, Mg, S, Na, Al, Fe, Mn, Zn, As, B, Cd, Co, Cr, Cu, Ni and Pb content, total nitrogen (TN), total organic carbon (TOC), cation exchange capacity (CEC) and assimilable phosphorus (AP)] of the rhizosphere soils of the 29 accessions of *S. dulcificum* studied had previously been determined by Adigoun et al. (2024) and were used in this study (S2 Table).

### Plant material surface disinfection

For surface disinfection, leaf and root subsamples were thoroughly washed three times with deionized water, immersed for 30 s in 1% NaClO solution supplemented with 0.1% Triton x100, washed for 15 s with EtOH (96%) and rinsed five times with sterile deionized water. A PCR was performed on the last rinsing water to confirm the surface disinfection of the samples [44]. The PCR was designed to amplify the bacterial 16S rRNA gene using the following universal primers: 27f (5′-AGA GTT TGA TCA TGG CTC A-3′) and 1492r (5′-TAC GGT TAC CTT GTT ACG ACT T-3′) (Eurofins Genomics, Paris, France) [45] and the DreamTaq™ Green PCR Master Mix (2x) kit (Thermo Fisher Scientific, Carlsbad, California). The PCR mix was prepared using 25 μl of Dream Taq Green master mix. Each primer was adjusted to 0.5 μM, 5 μl of the last rinsing water and the final volume was adjusted to 50 μl with nuclease-free water. DNA amplification was performed in a thermocycler (Mastercycler gradient, Eppendorf, Hamburg, Germany) under the following conditions: 95 °C for 2 min, 30 cycles at 95 °C for 30 s, 53 °C for 30 s, and 72 °C for 1 min, with additional 10 min at 72 °C. Bacterial DNA from a previously isolated bacterial strain was used as a positive control. Finally, leaf and root samples were considered successfully disinfected on the surface when no foreign DNA was detected after the PCR.

### DNA extraction and metagenomic analyses of endophytic bacterial communities

The root and leaf subsamples disinfected on the surface were frozen at −20 °C, freeze-dried and ground into a homogeneous powder under sterile conditions using a Mixer Mill for 45 s at 30 Hz (model MM400, Retsch Inc., Newtown, Pennsylvania, USA). The grinding was carried out after cutting the roots and leaves into small pieces in 2 ml tubes with 0.5 mm zirconium beads previously rinsed with 1% NaClO solution supplemented with 0.1% Triton x100 and EtOH (96%).

Subsequently, total metagenomic DNA was extracted using 100 mg of homogeneous powder of the plant material, with an adapted protocol based on hexadecyltrimethylammonium bromide (CTAB) chloroform alcohol [46]. Concisely, the DNA extraction protocol consisted of the combination of 1 h at 65 °C with multiple agitations in the CTAB buffer (CTAB 2% (w:v), EDTA 20 mM, Tris-HCl 100 mM, and NaCl 1.2 M), a heat shock (from −80 °C to 65 °C) and enzymatic digestion with proteinase K (~ 0.2 mg ml^-1^ per reaction), α-amylase from *Aspergillus oryzae* (~ 3 U ml^-1^ per reaction) and RNAse (~ 0.1 mg ml^-1^ per reaction). Thereafter, DNA precipitation was obtained with successive centrifugations (at 16,000 xg) in isopropanol (ambient temperature, 15 min) and ethanol 70% (4 °C, 30 min). The DNA extract was purified using a QIAquick® PCR purification kit (Qiagen, Germany). The purified DNA quantity and quality were assessed using a NanoDrop 2000c spectrophotometer (Thermo Fisher Scientific).

Equimolar DNA pools were adjusted to 10 ng/μl for PCR analyses. The PCR resulted in an amplicon of ~316 bp, appropriate for Illumina sequencing [47] after targeting the V5-V6 hypervariable regions of the 16S rRNA gene with the primers 799f (5′-AAC- MGGATTAGATACCCKG-3′) and 1115r (5′-AGG GTT GCG CTC GTT G-3′) [48,49] which exclude chloroplast DNA. Reactions were performed in three replicates for each sample with the following conditions: 3 min initial denaturation at 94 °C, followed by 35 cycles of 45 s at 94 °C, 30 s at 58 °C, and 90 s at 72 °C, with a final 10 min elongation at 72 °C. The resulting amplicons were purified with AMpure magnetic beads (Agencourt) and pooled in equimolar concentrations before sequencing with an Illumina MiSeq platform (ADNid, France). These libraries were mixed with Illumina-generated PhiX control libraries (5%) and sequenced with the MiSeq reagent kit V3-600 cycles (Illumina Inc., San Diego, USA).

### Bioinformatics and statistical analysis

Bacterial sequencing data were analyzed using the Mothur v.1.48.0 pipeline (last updated on 18/05/2022) [50]. Reads were assigned to each sample using a unique barcode and all raw read pairs were merged at the overlapping region V5-V6 of 16S rRNA gene. Contigs were then assigned and filtered by removing ambiguous sequences, homopolymers, artifacts and inappropriate length sequences. Sequences of the 16S rRNA gene considered as good quality and conserved were non-chimerical with at least 8 reads per sequence observed and had a minimal length of 200 bp and a maximum length of 500 bp.

The conserved sequences were subsequently clustered into Operational Taxonomic Units (OTUs) at 97% sequence similarity and were aligned with those present in the SILVA ribosomal RNA database v1.3.8 (16/12/2019) [51]. A Bayesian approach was used to perform taxonomic assignments [52] with the SILVA database. The Needleman distance was used to derive the OTUs and average neighbor clustering was done at a distance of 0.03. The Mothur ‘sub.sample’ function was used to rarefy the number of reads per sample to 3,547 representative sample sequences. Various alpha-diversity indices were estimated using the following functions with Mothur: ‘sobs’, ‘chao’, ‘shannon’, ‘shannoneven’, ‘invsimpson’ and ‘coverage’, to respectively estimate: observed OTU richness, Chao estimation of OTU richness [53], Shannon – Wiener diversity index, Shannon index-based measure of evenness, inverse Simpson diversity index and coverage, respectively. The coverage calculator returned a Good’s coverage OTU definition [54] and the coverage was calculated using the following equation: C = [1 − (n/N)] * 100 (%), where ‘n’ is the number of OTUs and ‘N’ the total number of sequences. The coverage helps to check whether the sequencing depth allowed a satisfactory representation of the bacterial communities in the samples. Alpha-diversity data were statistically analyzed across plant tissues and habitat types using the Wilcoxon signed-rank test on one hand and across phenotypic groups using the Kruskal-Wallis test on the other hand due to normality and homoscedasticity assumptions violation. The normality and homoscedasticity of the data were checked using the Shapiro and Levene’s tests respectively while the means separation was done using Dunn’s test [55] at the 0.05 probability level.

Non-metric multidimensional scaling (NMDS) plots were generated using the ‘meta- MDS’ function of the “vegan” R package [56] to study the beta diversity of the bacterial communities. An analysis of similarities (ANOSIM) was performed using the function ‘anosim’ in the vegan package to obtain R and p values indicating the strength of the tested factors on the samples and the significance levels, respectively. The relative abundances of endophytic bacteria by phyla/subphyla and orders in the samples across plant tissues, habitat types and phenotypic groups were represented using stacked bar charts with “ggplot2” package [57], while mean separations were done using Tukey’s test at the 0.05 probability level after a generalized linear model (glm) with a quasi-poisson-type error structure was performed to test the variation of OTU count. Sankey diagrams were generated to depict the relative abundance of the most abundant endophytic taxa from the leaves and roots of *S. dulcificum* accessions samples using “riverplot” package [58].

The relationship among the leaf and root mineral content variables and the accessions was assessed using a principal component analysis with the PCA function of the “FactoMineR” package [59]. A hierarchical clustering on principal components (HCPC) was used to group the accessions based on their mineral element contents and the results were presented using the fviz_cluster function in the R package “factoextra” [60]. The statistical difference among the means values of the clusters was tested using either an analysis of variance or Kruskal-Wallis test or t student or Wilcoxon rank test, when appropriate, followed by Tukey HSD test or Dunn’s test [55] at the 0.05 probability level for mean separation. Redundancy analysis (RDA) and Spearman correlations were performed to check the relationships between the leaf and root mineral contents and the relative abundance of the major bacterial phyla/class (>1%) for all accessions. Spearman correlations were also performed to reveal the link between the rhizosphere soil chemical characteristics and the relative abundance of the major endophytic bacterial phyla/class (>1%) of the accessions. Venn diagrams were used to identify the numbers of shared OTUs between roots and leaves, and among the phenotypic groups and habitat types for each plant tissue, and performed using the functions ggvenn and ggVennDiagram of the “ggvenn” and “ggVennDiagram” R packages [61,62]. The trans_diff function of the R package microeco [63] was used to perform the linear discriminant analysis effect size (LEfSe) [64] in order to highlight the taxonomical groups that are differentially abundant among the phenotypic groups. In addition, based on the 16S rRNA gene sequences, the potential functions of the endophytic bacterial communities were predicted using the Tax4Fun2 R package [65], which uses the KEGG (Kyoto Encyclopedia of Genes and Genomes) Orthology database for prokaryotes. The Wilcoxon rank test (p < 0.05) was performed to highlight significant differences between the relative abundance of the predictive gene profiles of KEGG level 2 and 3 pathways in the leaf and root endophytic bacterial communities.

All the data were statistically analyzed using R software version 4.3.1 [66].

## Results

### Chemical characteristics of *S. dulcificum* root and leaf samples

The mineral elements quantified at significant levels in the leaves of *S. dulcificum* by descending order of importance included: potassium (9,807 ± 382 mg kg^-1^), calcium (4,499 ± 181 mg kg^-1^), sulfur (2,373 ± 55 mg kg^-1^), magnesium (2,023 ± 62 mg kg^-1^) and phosphorus (1,267 ± 45 mg kg^-1^), while in the roots, minerals at high levels were calcium (13,350 ± 839 mg kg^-1^)> aluminum (8,834 ± 572 mg kg^-1^)> potassium (8,690 ± 513 mg kg^-1^)> sulfur (5,976 ± 229 mg kg^-1^)> magnesium (4,724 ± 170 mg kg^-1^). Overall, compared with the leaves, the roots of *S. dulcificum* were richer in all the elements except manganese, carbon, boron, nitrogen, and copper (Table 1).

**Table 1:** Descriptive statistics of mineral contents in leaves and roots of 29 *Synsepalum dulcificum* accessions in Benin.

Mineral	Minimum		Maximum		Mean		Standard error		Coefficient of variation	
	Leaf	Root	Leaf	Root	Leaf	Root	Leaf	Root	Leaf	Root
**N (g kg**^**-1**^)	13.60	4.90	26.00	15.30	18.52	8.11	0.30	0.17	14.71	19.43
**C (g kg**^**-1**^)	366.20	408.50	489.90	1,288.90	471.79	453.18	1.48	10.07	2.92	20.72
**P (mg kg**^**-1**^)	705.50	613.95	2,696.64	4,175.67	1,267.19	1,683.19	44.88	84.57	33.04	46.86
**K (mg kg**^**-1**^)	2,509.59	2,016.70	18,284.08	27,173.70	9,806.52	8,690.61	381.71	512.60	36.31	55.02
**Ca (mg kg**^**-1**^)	1,470.49	1,591.80	8,470.10	54,275.20	4,498.97	13,350.46	181.46	838.90	37.62	58.61
**Mg (mg kg**^**-1**^)	980.82	1,558.34	3,993.66	10,632.35	2,022.51	4,724.05	62.12	169.56	28.65	33.48
**S (mg kg**^**-1**^)	1,589.55	2,046.35	4,319.13	12,549.17	2,373.06	5,976.24	54.57	229.14	21.45	35.76
**Na (mg kg**^**-1**^)	179.54	81.68	9,647.92	9,610.03	1,172.38	2,169.05	175.39	243.88	139.54	104.88
**Al (mg kg**^**-1**^)	87.49	749.70	811.70	26,194.96	319.34	8,834.09	14.94	571.61	43.63	60.35
**Fe (mg kg**^**-1**^)	125.97	328.53	392.56	5,992.96	206.45	1,753.90	5.76	104.65	26.01	55.65
**Mn (mg kg**^**-1**^)	48.08	53.65	2,324.51	2,596.40	583.23	574.92	51.57	67.39	82.47	109.33
**Zn (mg kg**^**-1**^)	0.86	3.68	65.06	471.04	21.07	52.65	1.16	8.08	51.41	143.17
**As (mg kg**^**-1**^)	0.00	0.00	0.54	1.30	0.09	0.26	0.01	0.02	111.92	83.20
**B (mg kg**^**-1**^)	0.03	1.99	115.18	19.45	56.38	5.92	2.15	0.29	35.54	45.40
**Cd (mg kg**^**-1**^)	0.00	0.01	0.29	1.82	0.08	0.24	0.01	0.03	80.26	101.70
**Co (mg kg**^**-1**^)	0.00	0.00	1.11	7.09	0.05	0.59	0.02	0.11	362.04	187.07
**Cr (mg kg**^**-1**^)	0.00	0.15	1.80	4.09	0.49	1.31	0.04	0.08	68.11	62.29
**Cu (mg kg**^**-1**^)	0.00	2.10	107.19	19.02	7.70	6.03	1.18	0.34	142.92	52.72
**Ni (mg kg**^**-1**^)	0.00	0.51	10.27	55.63	2.04	4.21	0.20	0.65	89.76	144.42
**Pb (mg kg**^**-1**^)	0.05	0.20	0.71	6.69	0.34	1.08	0.01	0.11	37.66	98.37

C: total carbon, N: total nitrogen, P: phosphorus, K: potassium, Ca: calcium, Mg: magnesium, S: sulfur, Na: sodium, Al: aluminum, Fe: iron, Mn: manganese, Zn: zinc, As: arsenic, B: boron, Cd: cadmium, Co: cobalt, Cr: chromium, Cu: copper, Ni: nickel, Pb: lead.

**Fig 1:**
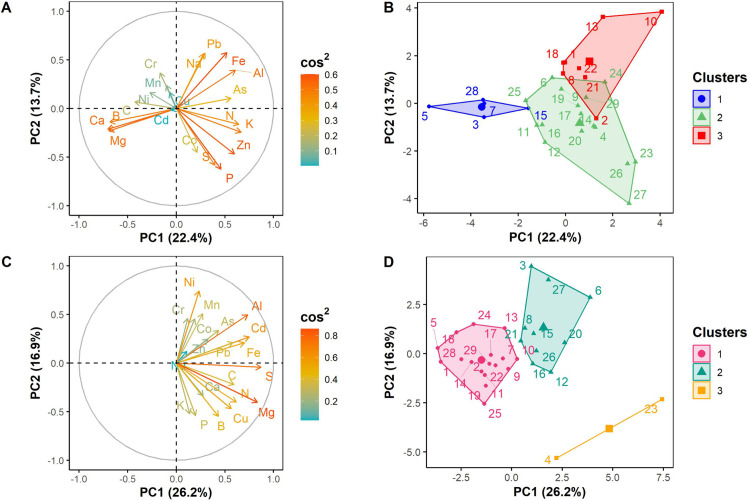
Principal component analysis with correlation circles (A, C) and factor maps (B, D) showing the grouping of the 29 *Synsepalum dulcificum* accessions based on 20 mineral element contents of the leaves (A, B) and the roots (C, D). The cos^2^ index shows the quality of representation of the parameters on the principal components.

Furthermore, the principal component analysis of the leaves’ mineral contents revealed that the first two components accounted for 36.1% of the total variation (Fig 1A). The first principal component explained 22.4% of the total variation and was significantly and positively correlated with leaf K, N, Al, Zn, As, Fe, P, and S contents, but negatively correlated with leaf C, B, Mg, and Ca contents. The second principal component explained 13.7% of the total variation and was significantly and positively correlated with leaves Fe, Pb, Na, Al, and Cr contents (Fig 1A). Hierarchical classification on the two principal components grouped the leaf samples of the 29 *S. dulcificum* accessions into three clusters with significant differences among them for most of the minerals studied (Fig 1B, Table 2). Cluster 1 included leaf samples from 5 accessions (17% of samples), characterized by significantly high levels of Ca, Mg and B. Cluster 2 is composed of leaf samples from 16 accessions (55% of samples) that were rich in P, K and S, while Cluster 3 included leaf samples from 8 accessions (28% of samples) with higher Na, Fe, Mn, and As contents (Fig 1B, Table 2). Moreover, the principal component analysis of the roots’ mineral contents showed that the first two components explained 43.1% of the total variation, with 26.2% and 16.9%, respectively (Fig 1C). The first principal component showed a significant and positive correlation with root S, Mg, Cd, Al, Fe, N, C, Pb, Cu, As, and B contents, while the second principal component was significantly and positively correlated with root Ni, Mn, Al, Cr, and Co contents, but negatively correlated with root N, Mg, Cu, K, P, and B contents (Fig 1C). In addition, the hierarchical classification, based on the first two principal components, grouped the samples into three clusters (Fig 1D). A significant difference was observed among these three clusters for the minerals studied (Table 2). The first cluster (Cluster 1) included root samples from 17 accessions (58.62% of samples) characterized by low levels of all the minerals. Cluster 2 comprised root samples (34.48% of samples) that exhibited high S, Al, Fe, Mn, Zn, Co, Ni, and Pb contents, while Cluster 3 comprised root samples (6.89% of samples) rich in N, C, K, Mg, S, B, and Cu (Table 2).

**Table 2:** Clusters’ descriptors for mineral contents in leaves and roots of 29 accessions of *Synsepalum dulcificum* in Benin.

Mineral	Leaf					Root				
	Cluster 1 (n = 5)	Cluster 2 (n = 16)	Cluster 3 (n = 8)	Significance#	Overall mean (n = 29)	Cluster 1 (n = 17)	Cluster 2 (n = 10)	Cluster 3 (n = 2)	Significance#	Overall mean (n = 29)
**N (g kg**^**-1**^)	17.13 ± 0.99	19.14 ± 0.66	18.14 ± 0.69	ns	18.51 ± 0.45	*7.69 ± 0.19 b*	8.28 ± 0.32 b	**10.7 ± 0.4 a**	***	8.11 ± 0.21
**C (g kg**^**-1**^)	476.19 ± 3.49	472.91 ± 1.37	*466.8 ± 3.74*	ns	471.79 ± 1.49	441.15 ± 2.48 b	442.13 ± 1.54 b	**610.7 ± 173.57 a**	***	453.18 ± 11.92
**P (mg kg**^**-1**^)	*960.29 ± 59.52 b*	**1,499.61 ± 91.62 a**	*994.15 ± 31.71 b*	**	1,267.19 ± 70.83	1,566.5 ± 154.58	1,730.31 ± 266.58	2,439.4 ± 594.86	ns	1,683.19 ± 135.92
**K (mg kg**^**-1**^)	*5,204.54 ± 1,091.06 c*	**11,738.71 ± 532.84 a**	8,818.39 ± 900.64 b	***	9,806.52 ± 618.68	8,104.62 ± 831.51	8,302.89 ± 1,012.58	**15,610.13 ± 9,841.9**	ns	8,690.61 ± 843.09
**Ca (mg kg**^**-1**^)	**5,967.24 ± 476.56 a**	4,341.54 ± 402.51 ab	3,896.15 ± 368.58 c	*	4,498.97 ± 283.18	13,581.40 ± 1,532.29	12,611.37 ± 1,063.18	15,083.00 ± 1,248.29	ns	13,350.46 ± 964.81
**Mg (mg kg**^**-1**^)	**2,602.9 ± 228.39 a**	2,037.82 ± 107.78 b	*1,629.13 ± 128.86 c*	**	2,022.51 ± 97.3	*4,090.78 ± 199.61 c*	5,045.63 ± 259.17 b	**8,498.89 ± 714.49 a**	***	4,724.05 ± 258.49
**S (mg kg**^**-1**^)	2,083.06 ± 123.59 b	**2,587.81 ± 114.12 a**	2,124.81 ± 80.8 b	**	2,373.06 ± 81.91	*4,976.52 ± 321.66 b*	**7,042.45 ± 297.66 a**	**9,142.73 ± 1,085.4 a**	***	5,976.24 ± 327.11
**Na (mg kg**^**-1**^)	401.94 ± 67.1 b	701.6 ± 76.36 ab	**2,595.48 ± 933.08 a**	**	1,172.38 ± 299.76	2,239.06 ± 574.4	1,953.91 ± 629.63	2,649.64 ± 551.15	ns	2,169.05 ± 395.54
**Al (mg kg**^**-1**^)	*191.85 ± 28.83 b*	336.76 ± 18.44 a	364.19 ± 51.48 a	*	319.34 ± 20.73	*6,571.54 ± 783.66 b*	**12,364.92 ± 831.8 a**	10,411.55 ± 3,740.41 ab	***	8,834.09 ± 764
**Fe (mg kg**^**-1**^)	*156.64 ± 5.56 b*	209.3 ± 6.05 a	**231.89 ± 20.33 a**	**	206.45 ± 7.87	*1,381.2 ± 143.57 b*	**2,317.52 ± 157.75 a**	2,103.69 ± 645.17 ab	***	1,753.9 ± 133.45
**Mn (mg kg**^**-1**^)	769.75 ± 116.19 ab	*366.33 ± 74.37 b*	**900.47 ± 211.34 a**	*	583.23 ± 84.74	*365.49 ± 72.44b*	**976 ± 242.08a**	349.77 ± 168.14b	*	574.92 ± 106.52
**Zn (mg kg**^**-1**^)	13.76 ± 2.46	22.92 ± 2.99	21.93 ± 2.4	ns	21.07 ± 1.89	*31.98 ± 6.89c*	**90.3 ± 25.6a**	40.1 ± 15.6b	*	52.65 ± 10.77
**As (mg kg**^**-1**^)	*0.02 ± 0.01 c*	0.09 ± 0.01 b	**0.13 ± 0.02 a**	***	0.09 ± 0.01	0.23 ± 0.03	0.32 ± 0.06	0.21 ± 0.11	ns	0.26 ± 0.03
**B (mg kg**^**-1**^)	**76.85 ± 6 a**	55.9 ± 3.87 b	*44.56 ± 2.86 b*	**	56.38 ± 3.14	5.54 ± 0.47b	5.65 ± 0.4b	**10.52 ± 1.5a**	**	5.92 ± 0.39
**Cd (mg kg**^**-1**^)	0.10 ± 0.04	*0.06 ± 0.01*	0.10 ± 0.02	ns	0.08 ± 0.01	*0.16 ± 0.02 b*	0.32 ± 0.05 ab	0.47 ± 0.36 a	*	0.24 ± 0.03
**Co (mg kg**^**-1**^)	0.04 ± 0.04	0.07 ± 0.06	0.01 ± 0.01	ns	0.05 ± 0.03	0.26 ± 0.04 b	**1.38 ± 0.48 a**	0.19 ± 0.09 b	**	0.59 ± 0.11
**Cr (mg kg**^**-1**^)	0.57 ± 0.08	0.44 ± 0.08	0.53 ± 0.06	ns	0.49 ± 0.05	1.07 ± 0.12 b	1.73 ± 0.17 a	1.52 ± 0.13 ab	*	1.31 ± 0.08
**Cu (mg kg**^**-1**^)	7.51 ± 0.89	8.55 ± 2.14	6.1 ± 0.67	ns	7.7 ± 1.2	*5.03 ± 0.36 b*	6.33 ± 0.57 b	**13.07 ± 0.55 a**	***	6.03 ± 0.48
**Ni (mg kg**^**-1**^)	**3.48 ± 0.88**	1.78 ± 0.47	1.66 ± 0.28	ns	2.04 ± 0.33	*2.74 ± 0.32 b*	**7.23 ± 1.8 a**	1.66 ± 0.6 b	**	4.21 ± 0.76
**Pb (mg kg**^**-1**^)	0.32 ± 0.04	0.32 ± 0.02	**0.40 ± 0.04**	ns	0.34 ± 0.02	*0.65 ± 0.06 b*	**1.78 ± 0.4 a**	1.22 ± 0.57 ab	**	1.08 ± 0.17

#: Significance was tested using Anova or Kruskal-Wallis test. n represents the number of accessions.

***, **, *indicate significance at the 0.001, 0.01, and 0.05 probability levels, respectively. ns: non-significant at p > 0.05.

Values (mean ± standard error) within a row followed by the different letters are significantly different according to Tukey’s HSD or Dunn’s post-hoc test at p < 0.05.

Clusters’ means significantly lower and greater than the overall means of all accessions are in bold, and italic, respectively, and best describe the given cluster.

C: total carbon, N: total nitrogen, P: phosphorus, K: potassium, Ca: calcium, Mg: magnesium, S: sulfur, Na: sodium, Al: aluminum, Fe: iron, Mn: manganese, Zn: zinc, As: arsenic, B: boron, Cd: cadmium, Co: cobalt, Cr: chromium, Cu: copper, Ni: nickel, Pb: lead.

### Endophytic bacterial community in the leaves and the roots of *S. dulcificum*

#### Endophytic bacterial community structure.

The bacterial 16S rRNA gene Illumina sequencing generated a total of 9,274,345 raw sequences from the 174 leaf and root samples collected. After quality assessment and filtering of chimeric, artifactual, unaligned, chloroplast and mitochondrial sequences, 2,487,732 sequences were obtained and then clustered into 2,468 OTUs at a 97% similarity threshold.

**Fig 2:**
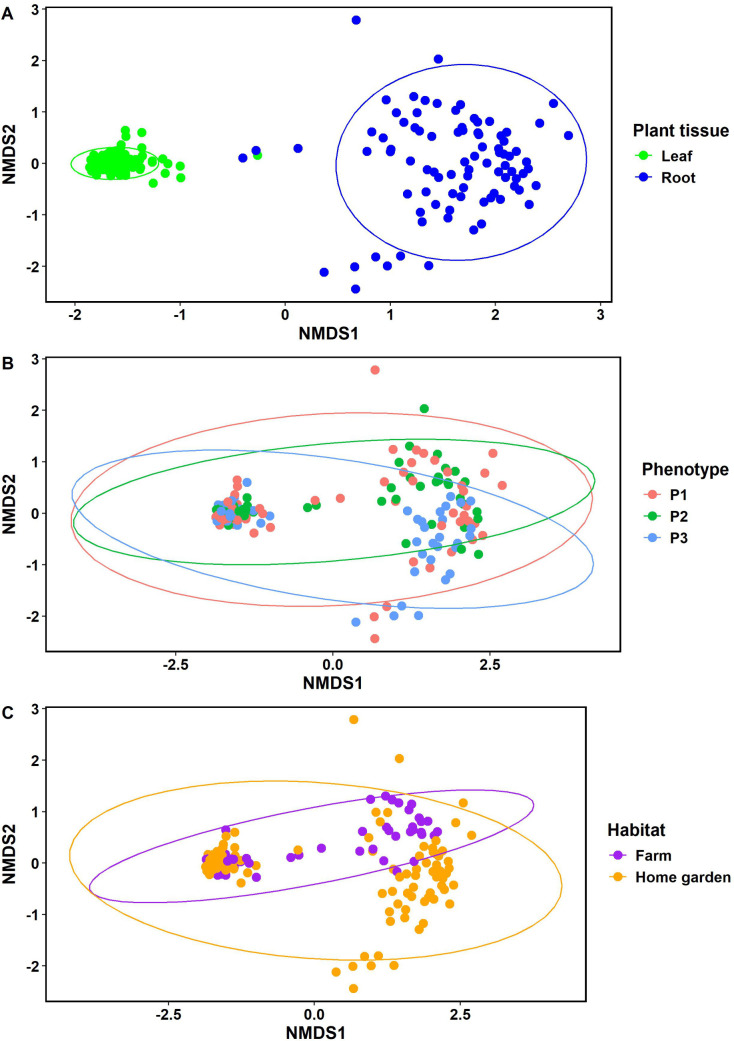
Non-metric multidimensional scaling (NMDS) plots of endophytic bacterial communities at OTUs level calculated with the method of Bray Curtis according to the plant tissue (A), phenotype (B) and habitat type (C). Each sample is represented by a point and each point color corresponds to the plant tissue type, phenotypic group (P1: group 1, P2: group 2, P3: group 3) or habitat to which the accessions sampled belong. The confidence area of the ellipses was 0.95 and the representation stress was equal to 0.102.

The beta-diversity of the endophytic bacterial communities was assessed using three non-metric multidimensional scaling (NMDS) plots (Fig 2). The first NMDS was used to check the similarity of the bacterial communities according to the plant tissue (leaf or root) and revealed a clear differentiation of the communities of the leaf samples from those of the roots of *S. dulcificum* (Fig 2A). The analysis of similarities (ANOSIM) further confirmed that endophytic bacterial communities in the leaves were significantly different (R = 0.75, p = 0.001) from those in the roots (Table 3). Conversely, the second and third NMDS plots revealed no differentiation among the bacterial communities of the three phenotypic groups (Fig 2B), as well as no differentiation between the bacterial communities of accessions located in farms from the ones in home gardens (Fig 2C). However, when considering samples from the leaves alone, the ANOSIM revealed a significant difference (R = 0.12, p = 0.016) between bacterial communities of accessions located in farms from the ones in home gardens (Table 3). In addition, in the roots, a significant dissimilarity (R = 0.03, p = 0.038) was observed among bacterial communities according to the phenotype of the accessions sampled (Table 3). Furthermore, the ANOSIM revealed that the accession from which samples were collected was the best factor that explained dissimilarities between the endophytic bacterial communities in the leaves (R = 0.31, p = 0.001) and the roots (R = 0.69, p = 0.001) when considered separately (Table 3).

**Table 3:** Analysis of similarities (ANOSIM) of the endophytic bacterial communities according to the plant tissue, phenotypic group, habitat and accession.

Factor tested		R-value	p-value	Figure
**Plant tissue**		0.7492	0.001	2A
**Phenotypic group**		−0.0068	0.751	2B
**Habitat type**		−0.0566	0.991	2C
**Accession**		0.0073	0.336	–
**Leaf**	**Phenotypic group**	0.0044	0.332	–
	**Habitat type**	0.1202	0.016	–
	**Accession**	0.3124	0.001	–
**Root**	**Phenotypic group**	0.0330	0.038	–
	**Habitat type**	0.0840	0.053	–
	**Accession**	0.6874	0.001	–

#### Endophytic bacterial community diversity.

The diversity of the bacterial community in the leaf and root endosphere of *S. dulcificum* accessions was revealed using alpha-diversity indices (Table 4). Results showed that all the estimated alpha-diversity indices, representing species richness, diversity, and evenness, varied significantly between leaf and root samples (Wilcoxon rank test, p < 0.05). The number of observed OTUs, Chao1 estimator, Shannon-Wiener, inverse Simpson and Shannon evenness indices were found to be significantly greater in the roots than in leaves. The average Good’s coverage was 99% and 97% in the leaves and the roots, respectively, suggesting a significant sequencing depth of the samples with a very low probability of unmeasured sequences. The richness, diversity and evenness of endophytic bacterial communities in the leaves were not significantly different across the accessions (Kruskall-Wallis test, p < 0.05), the phenotypic groups (Kruskall-Wallis test, p < 0.05) and habitat types (Wilcoxon rank test, p < 0.05) (Tables 4 and S3). Conversely, in the roots, a significant difference was observed across the accessions (Kruskall-Wallis test, p < 0.05) and across the phenotypic groups (Kruskall-Wallis test, p < 0.05) for the number of observed OTUs and the Chao1 estimator index, with accessions from the phenotypic group 3 revealing greater species richness than accessions from the phenotypic groups 1 and 2 (Tables 4 and S3).

**Table 4:** Alpha-diversity indices of endophytic bacterial communities associated with *Synsepalum dulcificum* according to the plant tissue and accessions’ phenotypic group and habitat type.

Factor		Modality	Observed OTUs	Chao1 estimator	Shannon-Wiener Index H’	Shannon Index Evenness E	Inverse Simpson Index1/D	Good’s coverage
**Plant tissue**		**Leaf**	52.59 ± 2.06 b	88.04 ± 3.82 b	1.05 ± 0.02 b	0.27 ± 0.00 b	1.68 ± 0.02 b	0.99 ± 0.00 b
		**Root**	246.71 ± 6.18 a	379.08 ± 10.52 a	3.35 ± 0.06 a	0.61 ± 0.01 a	11.48 ± 0.67 a	0.97 ± 0.00 a
**Leaf**	**Phenotypic group**	**Group 1**	54.27 ± 3.27 a	86.51 ± 5.12 a	1.07 ± 0.04 a	0.27 ± 0.01 a	1.69 ± 0.03 a	0.99 ± 0.00 a
		**Group 2**	50.81 ± 3.94 a	88.88 ± 8.46 a	1.03 ± 0.04 a	0.27 ± 0.01 a	1.67 ± 0.04 a	0.99 ± 0.00 a
		**Group 3**	52.3 ± 3.67 a	89.06 ± 6.63 a	1.05 ± 0.03 a	0.27 ± 0.01 a	1.68 ± 0.04 a	0.99 ± 0.00 a
	**Habitat type**	**Farm**	56.96 ± 4.35 a	95.74 ± 8.21 a	1.11 ± 0.04 a	0.28 ± 0.01 a	1.74 ± 0.04 a	0.99 ± 0.00 a
		**Home garden**	50.62 ± 2.24 a	84.57 ± 4.09 a	1.03 ± 0.02 a	0.26 ± 0.00 a	1.65 ± 0.02 b	0.99 ± 0.00 a
**Root**	**Phenotypic group**	**Group 1**	226.88 ± 10.74 b	347.1 ± 17.04 b	3.22 ± 0.11 a	0.59 ± 0.02 a	10.77 ± 1.16 a	0.97 ± 0.00 b
		**Group 2**	248.70 ± 10.27 ab	391.53 ± 18.70 ab	3.3 ± 0.12 a	0.6 ± 0.02 a	10.43 ± 0.93 a	0.97 ± 0.00 ab
		**Group 3**	268.96 ± 9.53 a	405.72 ± 17.66 a	3.55 ± 0.08 a	0.64 ± 0.01 a	13.42 ± 1.29 a	0.97 ± 0.00 a
	**Habitat type**	**Farm**	240.19 ± 9.37 a	381.45 ± 15.19 a	3.29 ± 0.09 a	0.6 ± 0.01 a	10.68 ± 1.05 a	0.97 ± 0 a
		**Home garden**	249.65 ± 7.93 a	378.01 ± 13.72 a	3.37 ± 0.08 a	0.61 ± 0.01 a	11.85 ± 0.85 a	0.97 ± 0 a

All diversity statistics were performed using an OTU threshold of ≥ 97% sequence similarity on randomly sub-sampled data at the lower sample size (3,547 reads). For each factor, values (mean ± standard error) within a column followed by the different letters are significantly different according to Dunn’s post-hoc test at p < 0.05.

#### Endophytic bacterial community composition.

**Fig 3:**
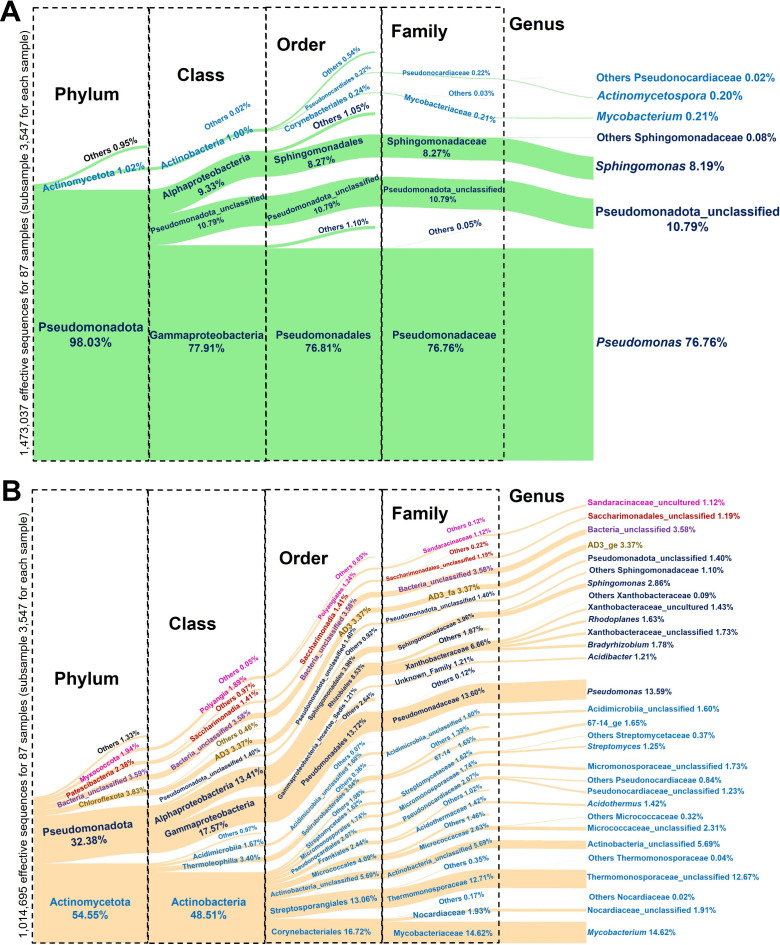
Visual depiction of the relative abundance of the dominant endophytic bacterial taxa from the leaves (A) and roots (B) of *Synsepalum dulcificum* through Sankey diagrams. The rarest taxa were not shown and grouped together in ‘Others’. The cut-off to classify an OTU as ‘Others’ was set at 1% relative abundance for all taxa.

The 2,468 OTUs recorded in the leaf and root endosphere of *S. dulcificum* were affiliated with a total of 20 bacterial phyla, 49 classes, 125 orders, 217 families, and 365 genera. The Sankey diagrams (Fig 3) showed that only two main bacterial phyla colonized the leaves against six main bacterial phyla in the roots. Leaf phyla included Pseudomonadota (synonym Proteobacteria), the most dominant with an average relative abundance of 98%, followed by Actinomycetota (synonyms Actinobacteria/Actinobacteriota, relative abundance of 1%) (Fig 3A). As for bacterial phyla of the root, they included by descending order of prominence: Actinomycetota (54.55%), Pseudomonadota (32.38%), Chloroflexota (synonym Chloroflexi, 3.83%), unclassified Bacteria (3.59%), Patescibacteria (2.38%) and Myxococcota (1.94%) (Fig 3B). The main genera recorded in the leaf endophytic bacterial communities included *Pseudomonas* (76.76%) from Gammaproteobacteria class and *Sphingomonas* (8.19%) from the Alphaproteobacteria class (Fig 3A). Among the root endophytic bacterial communities, 23 genera recorded a relative abundance greater than 1%. Thus, *Mycobacterium* (14.62%), *Acidothermus* (1.41%) and *Streptomyces* (1.25%) were among the genera found belonging to Actinobacteria class. From Gammaproteobacteria class, the genera observed included *Pseudomonas* (13.59%) and *Acidibacter* (1.21%). The bacterial community from Alphaproteobacteria class comprised the genera *Sphingomonas* (2.87%), *Bradyrhizobium* (1.78%) and *Rhodoplanes* (1.63%) (Fig 3B). Uncultured Sandaracinaceae (1.12%) and unclassified Saccharimonadales (1.19%) were the only genera recorded in the phyla Myxococcota and Patescibacteria, respectively.

**Fig 4:**
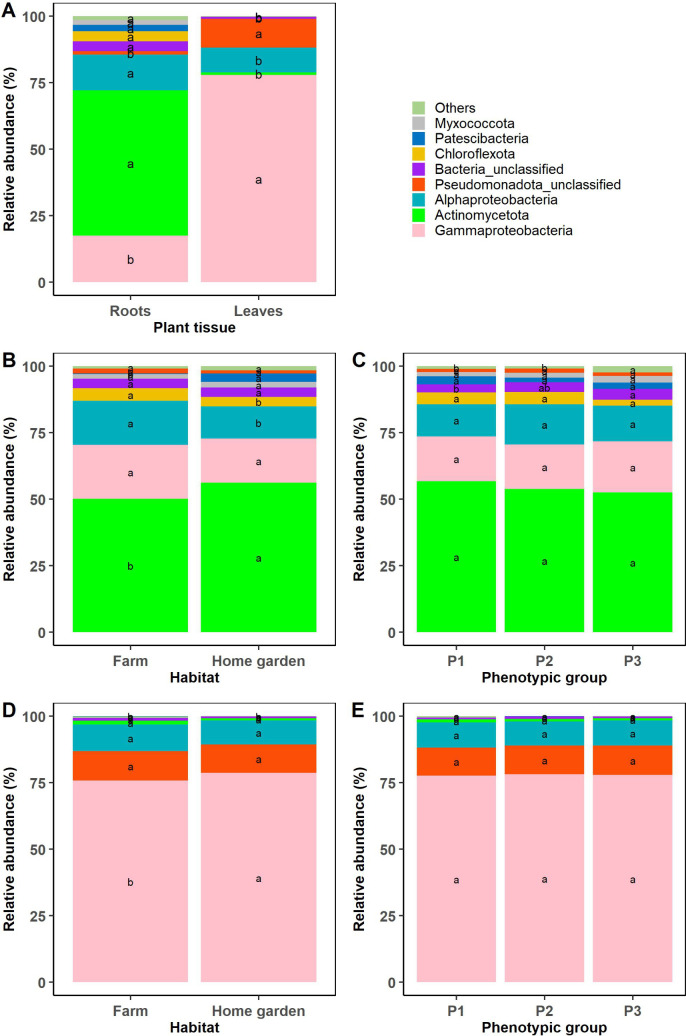
Relative abundance (%) of the major endophytic bacterial phyla/classes identified in the 29 accessions of *Synsepalum dulcificum* comparing the roots and leaves (A), the phenotypic groups in the leaves (C) and in the roots (E) (P1: n = 11, P2: n = 9, P3: n = 9), and the habitat types in the leaves (B) and in the roots (D) (farms: n = 9, home gardens: n = 20). “Others” (< 1%) were rare phyla and referred to Acidobacteriota, Armatimonadota, Bacteroidota, Bdellovibrionota, Deinococcota, Desulfobacterota, Elusimicrobiota, Entotheonellaeota, Bacillota, Gemmatimonadota, NB1-j, Nitrospirota, Planctomycetota, Verrucomicrobiota. A color was attributed to each phylum/class, and the values designated by the same letters were not significantly different (Tukey HSD test; p ≤ 0.05).

**Fig 5:**
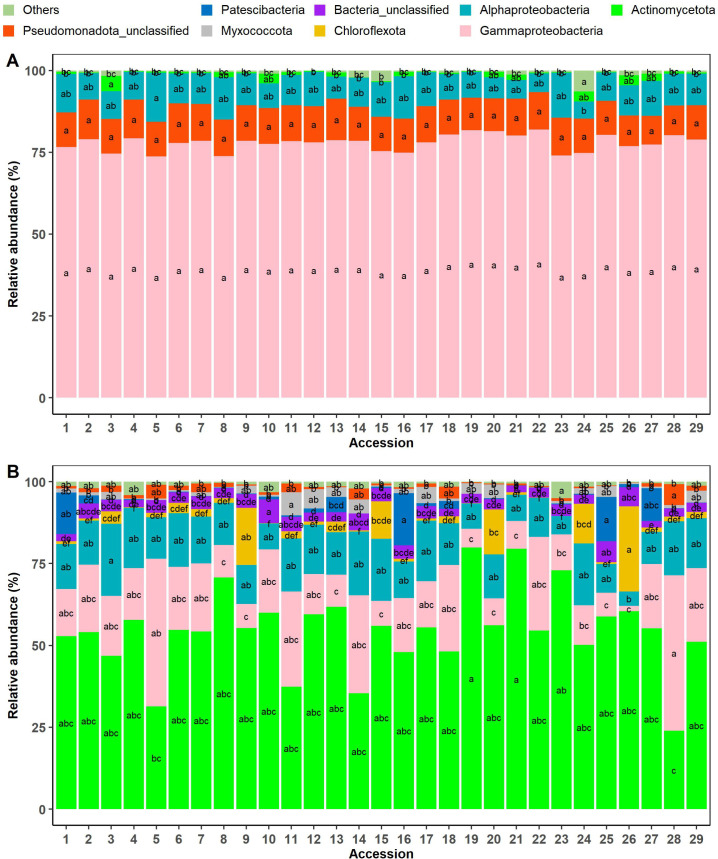
Relative abundance (%) of the major endophytic bacterial phyla/classes identified in the leaves (A) and roots (B) of 29 accessions of *Synsepalum dulcificum* sampled in Benin. “Others” grouped phyla with a relative abundance of less than 1% and referred to Acidobacteriota, Armatimonadota, Bacteroidota, Bdellovibrionota, Deinococcota, Desulfobacterota, Elusimicrobiota, Entotheonellaeota, Bacillota, Gemmatimonadota, NB1-j, Nitrospirota, Planctomycetota, and Verrucomicrobiota. A color was attributed to each phylum/class, and values designated by the same letters were not significantly different (Tukey HSD test; p ≤ 0.05).

Furthermore, the endophytic bacterial phyla/classes Actinomycetota, Alphaproteobacteria, Chloroflexota, unclassified Bacteria, Patescibacteria, and Myxococcota were significantly more abundant in the roots than in the leaves, while Gammaproteobacteria and unclassified Pseudomonadota classes were significantly more abundant in the leaves than in the roots (p < 0.05) (Fig 4A). When root endophytic bacterial communities were considered alone, Alphaproteobacteria and Chloroflexota were significantly more abundant in farms than in home gardens, while Actinomycetota and Patescibacteria were significantly more abundant in home gardens than farms (Fig 4B). However, considering the phenotypic groups, the unclassified and rare root endophytic bacteria (rare bacteria were regrouped in “Others” in the figure) were significantly more abundant in accessions from phenotypic group 3 (Fig 4C). On the other hand, in the leaf endophytic bacterial communities, Gammaproteobacteria class was highly abundant in accessions located in home gardens than in farms (Fig 4D), while no significant difference was observed across phenotypic groups (Fig 4E). Moreover, results revealed significant differences among accessions for the bacterial phyla and classes both in the leaves and the roots (Fig 5A and 5B). For example, in the leaves, Alphaproteobacteria and Actinomycetota were significantly abundant in accessions 5 and 3, respectively, while in the roots, these same taxa were highly abundant in accessions 3 and 19, 21, respectively. In the roots, accession 28 recorded the highest abundance of Gammaproteobacteria and unclassified Pseudomonadota, while accessions 16 and 25 recorded the greatest abundance of Patescibacteria (Fig 5B).

**Fig 6:**
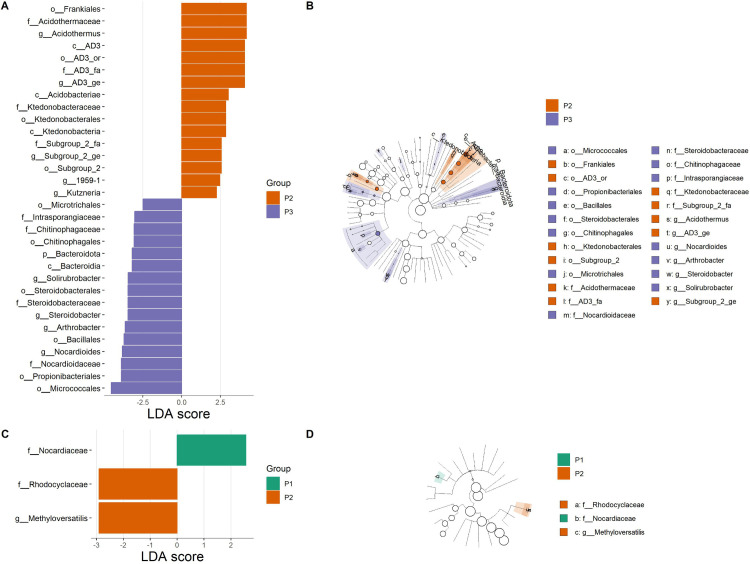
Linear discriminant analysis (LDA) for effect size (LEfSe) analysis of endophytic bacterial communities associated with *Synsepalum dulcificum* among the three phenotypic groups. LDA score histogram showing taxonomic groups that are statistically abundant for each group (LDA score = 2) in the roots (A) and the leaves (C). The degree of influence of the taxonomic groups is indicated by the length of the bar. Cladogram representing the phylogenetic structure of the identified taxa. In the cladogram, circles radiating from inside to outside show the classification (from phylum to genus) of each taxonomic group in the roots (B) and the leaves (D). Each small circle represents a taxonomic group and the diameter of the circle is proportional to the relative abundance. The taxonomic groups without significant differences were in white, and the identified biomarkers were colored in green for phenotypic group 1 (P1), red for phenotypic group 2 (P2), and purple for phenotypic group 3 (P3). p_: phylum, c_: class, o_: order, f_: family, g_: genus.

The LEfSe analysis showed significant differences in the abundance of the endophytic bacterial communities among the phenotypic groups at an LDA score > 2 (Fig 6). Thus, 32 taxonomic groups in the roots were differentially abundant among the phenotypic groups against three taxonomic groups in the leaves. Considering the root endophytic communities, the taxa identified as biomarkers were only related to the phenotypic groups 2 and 3, including the bacteria belonging to the genera *Acidothermus*, *Kutzneria*, *Solirubrobacter*, *Steroidobacter*, *Arthrobacter* and *Nocardioides* (Fig 6A and 6B). For the leaf communities, bacteria from the Nocardiaceae family were the only biomarker related to the phenotypic group 1, while the phenotypic group 2 recorded the Rhodocyclaceae family and the *Methyloversatilis* genus as the two biomarkers (Fig 6C and 6D).

### Mineral elements affecting the endophytic bacterial community composition in *S. dulcificum*

**Fig 7:**
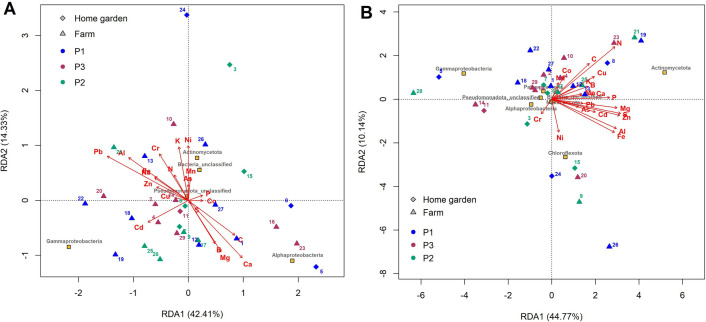
Redundancy Analysis (RDA) between the mineral element contents and the relative abundance of the major phyla/class of endophytic bacterial communities in the leaves (A) and roots (B) of *Synsepalum dulcificum* accessions. Accessions are colored according to the three phenotypic groups and the two habitat types under study. Diamond and triangle shapes represent home garden and farm habitats, respectively, while the blue, green and red colors correspond to the phenotypic groups 1 (P1), 2 (P2) and 3 (P3), respectively. The yellow square shape represents bacterial phyla/class.

The relationship between the relative abundance of the major bacterial phyla/class (>1%) found in *S. dulcificum* accessions from different habitat types and phenotypic groups and the mineral element concentrations in the leaves (Fig 7A) and in the roots (Fig 7B) was assessed using redundancy analysis (RDA). Results showed no signs of differentiation between accessions based on their habitat types or the phenotypic groups for both roots and leaves. In the RDA focused on the leaf sample characteristics (Fig 7A), 56.74% of the total variability was explained. The first axis, explaining 42.41% of the total variability, differentiated accessions based on Pb and Al contents (negative abscises). Axis 2 was negatively associated with the Mg and Ca contents and with the relative abundance of Alphaproteobacteria class. In contrast, this axis 2 was positively associated with Ni and K contents and with relative abundances of Actinomycetota phylum and unclassified Bacteria. The RDA focused on the root sample characteristics (Fig 7B), explained 54.91% of the total variability. The first axis explained 44.77% of the total variability and was positively associated with N and P contents and with the relative abundance of Actinomycetota phylum. Along this axis, accessions with root samples showing high mineral element contents were associated with a high relative abundance of Actinomycetota phylum and were differentiated from accessions characterized by low mineral element contents and a high abundance of Gammaproteobacteria class. Axis 2, which accounted for 10.14% of the total variation, differentiated accessions with higher Ni content and high relative abundance of Chloroflexota phylum (Fig 7B).

Spearman correlations (p < 0.05) were performed between the relative abundance of the endophytic bacterial phyla/class and the chemical characteristics in the leaves and roots for all the accessions, revealing moderately significant correlations among the parameters. Alphaproteobacteria class abundance in the leaves was positively correlated with the leaves’ Ca content but negatively correlated with the leaves’ Al and Pb contents (r = 0.39, r = −0.39 and r = −0.43, respectively). Gammaproteobacteria and unclassified Bacteria abundance in the leaves were positively correlated with leaf Na and Zn contents, respectively (r = 0.37 and r = 0.38, respectively). A negative correlation was observed between unclassified Bacteria and leaf Ca and Mg contents (r = −0.40 and r = −0.44, respectively). Regarding the root samples, Actinomycetota phylum abundance was positively correlated with root N, Mg, S, Cu, and Zn contents (r = 0.44, r = 0.50, r = 0.45, r = 0.39 and r = 0.57, respectively). A positive correlation was observed between Chloroflexota phylum and unclassified Bacteria abundance and root Ni (r = 0.67) and Ca (r = 0.52) contents, respectively. Moreover, negative correlations were found between bacterial classes belonging to the Pseudomonadota phylum and root mineral contents. Indeed, Alphaproteobacteria class abundance was negatively correlated with root Na and Zn contents (r = −0.37 and r = −0.54, respectively), Gammaproteobacteria class abundance was negatively correlated with root Fe, Mg and Zn contents (r = −0.38, r = −0.48 and r = −0.38, respectively), while unclassified Pseudomonadota abundance was negatively correlated with root Mg, Cu and Zn contents (r = −0.40, r = −0.44 and r = −0.52, respectively). Myxococcota phylum abundance in the roots was negatively correlated with the root N (r = −0.40) and Mn contents (r = −0.52).

**Fig 8:**
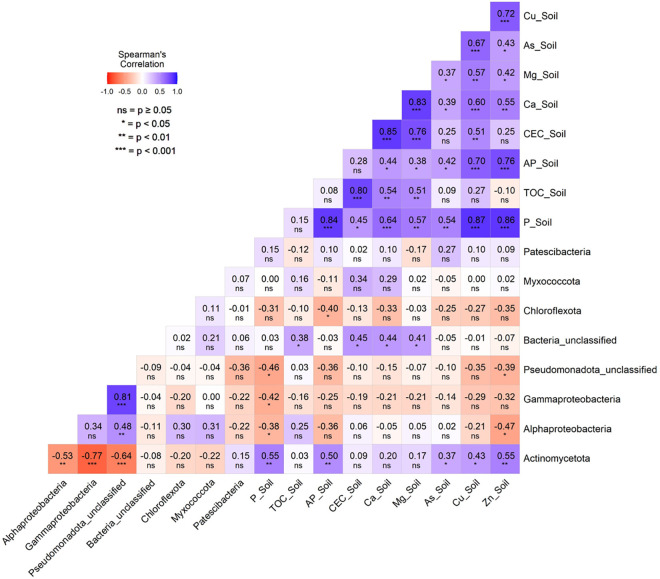
Correlation matrix depicting significant (p ≤ 0.05) Spearman correlations between the relative abundance of root endophytic bacterial major phyla/class and the chemical characteristics of the rhizosphere soils of the *Synsepalum dulcificum* accessions studied. The chemical characteristics of the rhizosphere soils included total organic carbon (TOC_Soil), phosphorus (P_Soil), assimilable phosphorus (AP_Soil), cation exchange capacity (CEC_Soil), calcium (Ca_Soil), magnesium (Mg_Soil), zinc (Zn_Soil), arsenic (As_Soil), and copper (Cu_Soil).

Furthermore, the Spearman correlations (p < 0.05) performed between the chemical characteristics of the rhizosphere soils associated with the *S. dulcificum* accessions and the relative abundance of the endophytic bacterial phyla/class revealed no significant correlation with the leaf community but significant correlations with the community in the roots (Fig 8). Therefore, in the root endophytic bacterial community, Actinomycetota phylum abundance was positively correlated with the rhizosphere soil phosphorus (P and assimilable phosphorus content) and metal contents (As, Cu and Zn), while unclassified Bacteria abundance was positively correlated with the soil capacity exchange cation, total organic carbon, Ca and Mg content. Conversely, negative correlations were found between the abundance of Pseudomonadota classes (Alphaproteobacteria, Gammaproteobacteria, and unclassified Pseudomonadota) and the soil P and Zn contents. Chloroflexota abundance was also negatively correlated with soil-assimilable phosphorus content (Fig 8).

### Major OTUs shared in the leaf and root endophytic bacterial communities of *S. dulcificum*

**Fig 9:**
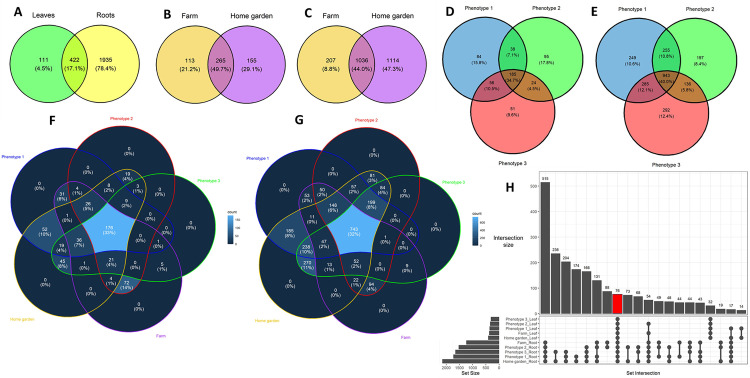
Number of shared and specific OTUs of endophytic bacterial communities according to the type of plant tissue (A), the three phenotypic groups (B: leaves, C: roots) and the two habitat types (D: leaves, E: roots) in *Synsepalum dulcificum.* Venn diagrams (F) and (G) show the number of shared and specific OTUs across the combination of phenotypic groups and habitat types in the leaves and roots, respectively. OTU-level upset diagram depicting the number of shared OTUs combining the type of plant tissue, the phenotypic groups and habitat types (H). The set size refers to the number of OTUs in the samples considering each factor, and the intersection size represents the number of OTUs shared for each combination of factors. The barplot in red shows the number of OTUs shared by combining all the factors.

The analysis of species richness within endophytic bacterial communities revealed that 533 and 2,357 OTUs were recorded in the leaf and root samples studied, respectively, out of which 422 OTUs [representing 17% (422/2,468) of the total OTUs observed in the endophytic communities studied] were shared (Fig 9A). Considering the OTUs in each plant tissue, 185 and 265 OTUs were shared among the three phenotypic groups (group 1 *vs* group 2 *vs* group 3) (Fig 9D) and the two habitat types (farm *vs* home garden) (Fig 9B), respectively in the leaves, while 943 and 1,036 OTUs were shared among the three phenotypic groups (Fig 9E) and the two habitat types (Fig 9C), respectively in the roots. Thus, 35% (185/533) of the OTUs observed in the leaves were shared among phenotypic groups compared to 40% (943/2,357) in the roots. Conversely, more OTUs were shared between home garden and farm accessions in the leaves (50%) than in the roots (44%). Furthermore, the Venn diagrams revealed that in the leaves, 176 (33% of the OTUs in the leaves) OTUs were consistently present in samples from all three phenotypic groups and two habitat types (group 1 *vs* group 2 *vs* group 3 *vs* farm *vs* home garden) (Fig 9F) while 743 (32% of the OTUs in the roots) OTUs were recorded in the roots (Fig 9G). Also, 20 out of the 176 and 102 out of the 743 OTUs were identified as major OTUs in each plant tissue (relative abundance ≥ 0.1%). Finally, combining all the factors: plant tissues, phenotypic groups and habitat types, the upset plot revealed a total of 76 OTUs that were shared across the endophytic bacterial communities, which represented 3% (76/2,468) of the total OTUs found in the endophytic bacterial communities studied (Fig 9H). In addition, 8 out of the 76 OTUs shared had a relative abundance ≥ 0.1% in each plant tissue and could therefore be considered as major OTUs. These eight major OTUs were represented as follows: OTU0001 (genus *Pseudomonas*), OTU0003 (*Sphingomonas*), OTU0004 (*Mycobacterium*), OTU0008 (unclassified Pseudomonadota), OTU0009 (unclassified Pseudomonadota), OTU0010 (unclassified Pseudomonadota), OTU0011 (unclassified Pseudomonadota) and OTU0083 (*Methyloversatilis*).

### Potential functions of endophytic bacterial community in the leaves and roots of *S. dulcificum*

**Fig 10:**
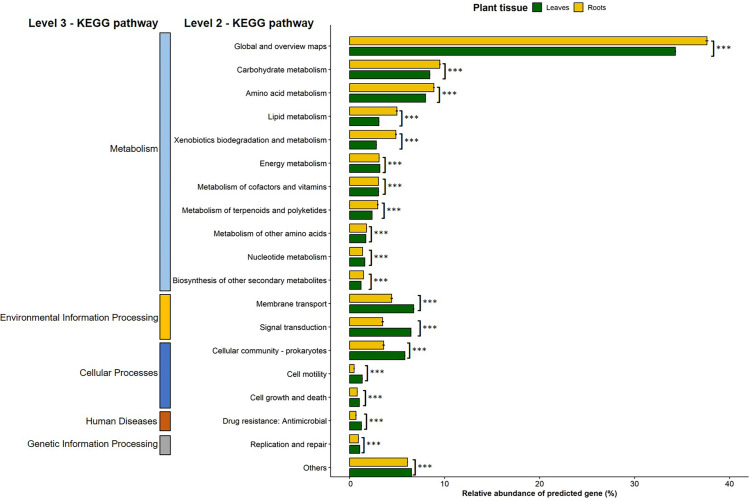
Gene profiles of leaf and root endophytic bacterial communities of *Synsepalum dulcificum* predicted using Tax4Fun 2. “Others” grouped gene profiles related to level 2 KEGG pathways with a relative abundance of less than 1%. *** indicate significance at the 0.001 probability level tested using the Wilcoxon rank test.

The Tax4Fun2 analysis of 16S rRNA data used to predict the functional profiles of the endophytic bacterial communities identified 4,831 KEGG orthologs (KO) in the leaves against 7,000 KEGG orthologs in the roots. These orthologs were classified into these six KEGG level 3 pathways: metabolism, environmental information processing, organismal system, genetic information processing, cellular processes and even human disease (S4 Table). Significant differences (p < 0.05) were found between the plant tissue (leaf and root) for the relative abundance of the level 3 KEGG pathways (S4 Table). Indeed, gene functions related to metabolism and organismal system pathways were significantly more abundant in the root than the leaf tissue. On the contrary, functions related to cellular processes, environmental information processing, genetic information processing, and human disease were significantly more abundant in the leaf than in the root tissue (S4 Table). Furthermore, 45 and 46 of the level 2 KEGG Orthology groups were recorded in the leaves and the roots, respectively, among which 18 had a relative abundance greater than 1% (Fig 10). Regardless of the type of plant tissue, the functional profile of the endophytic bacterial community of *S. dulcificum* indicated that the overall functional structure of the community was dominated by metabolism-related KEGG pathways, with the level 2 KO group named “global and overview maps” being the most dominant. The “global and overview maps” included level 1 KEGG pathways related to metabolic pathways, biosynthesis of secondary metabolites, microbial metabolism in diverse environments, biosynthesis of antibiotics, carbon metabolism, 2-oxocarboxylic acid metabolism, fatty acid metabolism, degradation of aromatic compounds, and biosynthesis of amino acids. Gene functions related to nucleotide, amino acid (arginine, alanine, glycine, cysteine, methionine, valine, leucine, phenylalanine, tryptophan…), carbohydrate (glycolysis, pyruvate, fructose, galactose, inositol phosphate, starch, sucrose…) energy (oxidative phosphorylation, photosynthesis, methane metabolism, carbon fixation pathways in prokaryotes, nitrogen metabolism, sulfur metabolism…), cofactor, vitamins and lipid metabolism were also recorded. Another dominant KEGG category of the predicted functional gene profile of the endophytic bacterial community is related to environmental information processing, especially pathways related to membrane transport and signal transduction. KO associated with the cellular community of prokaryotes and cell motility pathways were also recorded as part of cellular processes. Moreover, the relative abundance of the 18 major level 2 KEGG pathways was significantly different (p < 0.05) between the leaf and root endophytic bacterial communities (Fig 10). For instance, KO related to carbohydrate metabolism, lipid metabolism, amino acid metabolism, biosynthesis of other secondary metabolites, xenobiotics biodegradation and metabolism were significantly more abundant in the roots than in the leaves. Contrarily, KO related to membrane transport, signal transduction, cellular community of prokaryotes, cell motility, and cell growth and death were significantly more abundant in the leaves than in the roots (Fig 10).

## Discussion

### How diverse is the endophytic bacterial community associated with *S. dulcificum* across plant tissues, accessions, phenotypic groups and habitat types?

Beneficial endophytic bacteria interact with host plants and are essential regulators of their growth and resilience to environmental stresses [67]. Understanding the diversity and composition of plant-beneficial endophytic bacterial communities is, therefore, crucial for better harnessing their potential in each host plant [68]. As bacterial endophytic communities vary according to different plant factors [29], we hereby reported the endophytes associated with *Synsepalum dulcificum* from its native environment as the first step toward the use of endophytic bacteria to enhance the growth of the species known for its slow growth nature. *S. dulcificum* hosts a highly diversified community of bacterial endophytes, which are known to inhabit different plant tissues. This diversity varied across plant tissues with a reduction from root to leaves. Specifically, the number of observed OTUs, Chao1 estimator, Shannon-Wiener, inverse Simpson and Shannon evenness indices were significantly greater in the roots than in the leaves, indicating a higher species richness and community diversity in the roots than in the leaves of *S. dulcificum*. This could be related to the close interconnection of roots with the rich rhizosphere reservoir of microorganism communities [69]. Furthermore, the NMDS plot, coupled with the ANOSIM performed, revealed a clear differentiation between the communities of the leaf samples and those of the roots. The diversity recorded here was lower than the one our team previously reported on the rhizobacteria community of the same accessions [70]. It is, therefore, clear that the bacteria diversity decreased along with the rhizosphere-root-leaf continuum. This is well known in plant species as only specialized bacteria could colonize specific plant tissue. In addition, unlike the rhizosphere, endophytic bacteria are less prone to the influence of environmental factors [21]. Similar results were observed by Liu et al. [42], who focused on endophytic bacteria in all parts of the same species (leaf, root, stem, flower) in China, showing that roots harbor a more diversified community than leaves. Likewise, Sangiorgio et al. [32] recorded in their study on strawberry (*Fragaria x ananassa*) cultivars that above-ground organs had a lower bacterial richness compared to other compartments like bulk soil, rhizosphere and root, even when epiphytic bacteria were included in the root and above-ground organs communities. Moreover, the same tendency was recorded in sweetpotato (*Ipomoea batatas* (L.) Lam), tomato (*Solanum lycopersicum* L.) and African wild rice (*Oryza longistaminata* A. Chev. & Roehr.) plants where the diversity and species richness of endophytic bacteria were significantly higher in the roots than in the leaves [71–73]. Plant tissue characteristics represent, therefore, important regulators of microbial abundance, showing a wide variance in the richness and diversity of endophytes among plant tissues [74,75]. This also indicates the potential role that plant physiology may play in structuring endophytic communities [76,77].

Furthermore, when focusing on each endosphere niche separately, the ANOSIM revealed that the factor habitat type discriminated the bacterial communities in the leaves while the phenotypic groups discriminated the root bacterial communities, suggesting that these two factors influence the endophytic bacterial communities. It is nonetheless important to mention, based on the beta diversity analysis, that whether in the leaves or in the roots, the endophytic bacterial communities varied greatly across accessions of *S. dulcificum*. These results suggested that different environmental factors, including the habitat, phenotype and accession, affected the diversity of endophytic bacterial communities within species at different levels of importance. These same factors were previously reported to affect rhizosphere bacteria diversity in the same crop species [70]. In a review article, Papik et al. [77], summarized the factors affecting the endophytic bacteria structure into two types: (i) biotic factors including plant genotype, developmental stage and physiology, root exudation, secondary metabolites, cuticle properties, microbe-microbe interactions within the host plant and (ii) abiotic factors including soil type, presence of pollutants, moisture, pH, temperature and climate oscillations. Additionally, Xiao et al. [78] found that in watermelon (*Citrullus lanatus* L.), the fruit shape was affected by bacterial richness and composition through the recruitment of specific microorganisms in the rhizosphere and stem endosphere. Furthermore, comparing the leaf and root endophytic bacterial communities of seven banana genotypes, Singh et al. [79] showed that some key taxa and protective functions were affected by the plant genotype and might therefore contribute to differences in host resistance to banana pandemic diseases. Cultivar-dependent changes were also reported in the leaf endophytic bacterial diversity of grapevine suggesting that the host genotype has a key role in shaping its own bacterial microbiome [80].

In this study, a relatively low alpha diversity was observed in *S. dulcificum* compared to other crop species like blueberry [81], apple [82] and banana [79]. For instance, Singh et al. [79] recorded an average Shannon diversity value of 2.62 and 4.42 in banana leaf and root endosphere, respectively, compared to 1.05 and 3.35 in *S. dulcificum* leaf and root endosphere respectively in Benin. As the plant species is also a factor that could influence the endophytic bacterial diversity [83], the low alpha diversity observed in this study could be explained by the fact that only specialized endophytic bacteria could colonize *S. dulcificum* tissues due to different species related factors like the nature of the secondary metabolites and root exudates produced in the plant and environment studied [84].

Our results also highlighted that the most abundant phyla of bacteria in root tissues included Actinomycetota, Pseudomonadota, Chloroflexota, Patescibacteria, and Myxococcota and being Pseudomonadota and Actinomycetota in the leaf tissues. Some of these phyla were also associated with *S. dulcificum,* as shown by Liu et al. [42] in China. Indeed, these authors found Pseudomonadota, Actinomycetota, Bacillota (synonym Firmicutes), Cyanobacteria, Bdellovibrionota, and Bacteroidota phyla being commonly present in the species root and leaf tissues with Verrucomicrobiota and Planctomycetota recorded only in the roots. However, even though our results showed the presence of the Pseudomonadota phylum in both tissues (root and leaf), Liu et al. [42] reported that the Pseudomonadota phylum dominated in all the studied tissues, while in our study, this dominance was only observed in the leaves. The difference could be attributable to the environment and the genotype of the *S. dulcificum* plants sampled in their study since our results also found that the phenotype and accessions affected the diversity of endophytic bacteria communities observed. Moreover, our team previously found that Actinomycetota was the most abundant phyla in the species rhizosphere, and the same tendency was observed in the root tissues [70]. This could be explained by the fact that the host plants selected from the same phyla, the bacteria communities that could specifically colonize the root tissues that are closely linked to the rhizosphere. Pseudomonadota and Actinomycetota phyla were also the most abundant phyla found in other fruit species’ leaf and root tissues like strawberry [32], watermelon [78] and Amur grape (*Vitis amurensis* Rupr.) [85]. Similarly to the results of Liu et al. [42], we recorded Gammaproteobacteria, Alphaproteobacteria and unclassified Pseudomonadota as the bacterial classes belonging to the Pseudomonadota phylum. Thus, the relative abundance of these classes and the other phyla found was significantly different across plant tissues, accessions, phenotypic groups and habitat types. This confirmed the results of the beta diversity, showing that these factors also influence the endophytic bacterial taxonomic composition of *S. dulcificum*. Regarding the genera abundances, our results found *Pseudomonas* (76.76%) and *Sphingomonas* (8.19%) as the two majors in the leaves while *Mycobacterium* (14.62%) and *Pseudomonas* (13.59%) were the ones in the roots. On the contrary, even though these above-mentionned genera were recorded by Liu et al. [42], among the endophytes associated with the species in China, *Ralstonia* was the most dominant genus in the leaves (59.82%) and the roots (39.87%).

### How are *S. dulcificum* compartments’ chemical characteristics and endophytic bacterial taxa composition related?

Our results revealed, in the leaves, positive correlations between Alphaproteobacteria, Gammaproteobacteria, unclassified Bacteria abundance and Ca, Zn and Na contents but negative correlations between Alphaproteobacteria abundance and Al and Pb contents. This showed that high levels of Ca, Zn and Na were related to the abundance of these bacterial taxa in the leaves, while in contrast, the leaf metal (Al and Pb) contents were associated with a decreased abundance of Alphaproteobacteria. In the roots, the most dominant phylum (Actinomycetota) abundance was related to high concentrations of N, Mg, S, Cu, and Zn, while Pseudomonadota, the following was associated with low concentrations of Na, Zn, Mg, Fe, and Cu in the roots. Chloroflexota phylum was more abundant in Ni-enriched roots. Some of the correlations found in the roots were also highlighted in the results of Adigoun et al. [70], which showed that Chloroflexota abundance in *S. dulcificum* rhizosphere soils was positively correlated with the soil Al, As, Fe, and Ni contents. They also found in their study that the Actinomycetota abundance in the soil was positively correlated with the soil’s total nitrogen content. These similarities recorded might be due to the close relationship between the rhizosphere soils and the root endosphere, which favored the enrichment of these two niches of specific bacterial communities affected by the availability of different nutrients in each plant compartment (root and rhizosphere soil). Henning et al. [40] demonstrated that root endophytic bacterial community composition influenced the nitrogen and phosphorus concentration of *Populus deltoides* W. Bartram ex Marshall shoots and roots. Our results also found significant associations between the dominant bacterial taxa in the root tissue and the rhizosphere soil chemical characteristics, indicating the possible role of the rhizosphere in selecting the endophytic bacterial community in the root tissue of the plant species. For example, the abundance of the Actinomycetota community in the root tissue of *S. dulcificum* is associated with high P and low metal (As, Cu, Zn) content of the rhizosphere soil, while the abundance of Pseudomonadota in the same tissue was associated with low P and Zn content. In addition, Chloroflexota were abundant in the root tissue of rhizosphere soil showing low phosphorus content. Previous studies have also shown that the abundance of Actinomycetota communities was positively correlated with soil nitrogen content in agricultural fields [86] and tropical rainforest soils [87]. These communities are consequently considered as copiotrophic bacteria. Xu et al. [88] also demonstrated that endophytic bacterial communities in rice seedling roots were affected by soil P deficiency, with endophytic Pseudomonadota significantly increasing under initial P starvation in the soil. Moreover, Li et al. [89] used random forest modelling to reveal that mineral nitrogen in the soil was a significant predictor of endophytic bacterial community changes in sorghum [*Sorghum bicolor* (L.) Moench] root tissues. Orellana et al. [90] also argued that the plant responses to different bacterial strains belonging to the Pseudomonadota phylum were influenced by several factors, including the plant nutrient concentrations and availabilities, the elapsed time of the interaction, as well as the specific identities of the beneficial bacteria strain used. Regarding the metal concentrations, even though a previous study has shown that Actinomycetota, Pseudomonadota and Bacillota were the predominant phyla found in the associated root endophytic community of As-enriched soils [91], our results only found Actinomycetota positively associated with the soil As, Cu and Zn content. This could be explained by the fact that the soils studied are not metal-contaminated but contain a standard threshold of metal concentrations, as observed in agricultural soils [70]. The bacterial communities found in nutrient-enriched plant compartments (soil, roots, and leaves) could be involved in improving the nutritional status of *S. dulcificum* and, consequently, influence its growth.

### Does an endophytic bacterial core microbiome exhibiting plant growth-promoting traits exist in *S. dulcificum*?

Our results revealed that 17% of the total OTUs recorded in the endophytic bacterial communities studied were shared between the leaf and root endophytic bacterial communities. In addition, 4.5% (leaf) and 78.4% (root) of the total OTUs were specific to each plant tissue. These indicated that very few endophytic bacterial communities were able to move across the root-leaf continuum, with the leaves being very selective with the communities allowed in that niche. The variations in endophytic diversity and proportion of shared bacterial taxa among distinct plant tissues might be linked to different environmental sources of the endophytes and their capability to colonize different niches *in planta* [77]. More OTUs were shared in the leaves (50%) than the roots (44%) between the two habitat types. On the contrary, across phenotypic groups, more OTUs were shared in the roots (40%) than in the leaves (35%). Though the number of OTUs was four times more abundant in the roots than in the leaves, the percentage of OTUs shared was approximately the same across the combination of phenotypic groups and habitat types in the leaves (33%) and in the roots (32%). More importantly, among all these shared OTUs, only 76 OTUs (3%) were common to the endophytic bacterial communities when considering the plant tissue from which they were found, the habitat types, as well as the phenotypic groups of the accessions sampled. Most of these 76 OTUs were rare because they have a relative abundance of less than 0.1%. Eight OTUs were abundant with a relative abundance greater than 0.1% and considered major OTUs shared across the leaf and root endophytic bacterial communities associated with *S. dulcificum*. This core bacterial microbiome was identified as follows: OTU0001 (genus *Pseudomonas*), OTU0003 (*Sphingomonas*), OTU0004 (*Mycobacterium*), OTU0008 (unclassified Pseudomonadota), OTU0009 (unclassified Pseudomonadota), OTU0010 (unclassified Pseudomonadota), OTU0011 (unclassified Pseudomonadota), and OTU0083 (*Methyloversatilis*). Thus, four genera were represented in the core endophytic bacterial microbiome: *Pseudomonas*, *Sphingomonas*, *Mycobacterium* and *Methyloversatilis*. These genera included endophytic bacterial strains that were intensively reported to be positively involved in several plant functionalities [92,93] including nitrogen fixation in *Oryza sativa* [94], shoot growth in *Malus domestica* Borkh [95] and *Eleusine coracana* Gaertn [96], biodefense in *Beta vulgaris* L. (Zachow et al., 2015) and grain quality improvement in *E. coracan*a [96]. Moreover, *Sphingomonas* sp. LK1 improved soybean’s auxin (IAA) production, phosphate and trehalose metabolism [97]. Pan et al. [98] also demonstrated that *Mycobacterium* Mya-zh01 improved the orchid *Doritaenopsis* seed germination and growth. More importantly, the unclassified Pseudomonadota found among the OTUs could also contain bacterial strains that exhibit great plant-growth-promoting traits useful for improving *S. dulcificum* growth.

### What are the potential functions of the endophytic bacterial community of *S. dulcificum*?

Tax4Fun2, which provides a convenient method for predicting microbial communities’ functions, revealed the potential functions of the endophytic bacterial community of *S. dulcificum*. Thus, our results revealed common patterns of functional differences in the bacterial communities between the two plant tissue types, with functions related to metabolic pathways being the most abundant in the endophytic bacterial community of *S. dulcificum*. More generally, functions related to different metabolic pathways, including carbohydrates, amino acid, lipid, energy (nitrogen, methane and sulfur), cofactors and vitamins metabolism, were widely prevalent, showing the potential role of endophytic bacterial communities in the use of different sources of carbon and nitrogen to improve the host plants’ growth [34]. Furthermore, the gene profile prediction revealed that root bacterial communities could be more active in the metabolism of carbohydrates, lipids, and amino acid while the leaf bacterial community could be more involved in membrane transport, signal transduction, cell motility, growth and death. The enhanced ability of bacteria to metabolize carbohydrates in the roots may be due to the variability in the source of carbon and nitrogen available in the roots of *S. dulcificum* with different natures of exudates secreted in the rhizosphere. Chen and Liu [99] reported that different types of exudates secreted in the roots serve as nutrients, antimicrobial substances, or signals, facilitating root colonization by beneficial bacteria. The membrane transport and cell motility functions could enable bacteria to interact with their surroundings but also to move across different tissue types, also facilitating chemotactic responses to chemical gradients generated by rhizodeposits and other signals in the rhizosphere and the plant tissues [100]. In fact, the root exudates, which shape the chemical characteristics (mineral contents especially) of the rhizosphere, may be involved in recruiting bacterial endophytes from the rhizosphere as they also contain substrates that initiate early communication between bacterial endophytes and host plants [101]. In addition, the root endophytic bacterial community showed more genes involved in xenobiotics biodegradation and metabolism, indicating this community’s potential in degrading and transforming synthetic compounds (herbicides and pesticides, for example) and other environmental pollutants for bioremediation [102]. Interestingly, our results also highlighted that the endophytic bacterial community of *S. dulcificum* could exhibit functions related to the biosynthesis of secondary metabolites. Indeed, Liu et al. [42] demonstrated that some endophytes in *S. dulcificum* from Xishuangbanna (China) have the potential to produce active secondary metabolites. The endophytic bacteria communities in our study could be harnessed for secondary metabolites biosynthesis for the production of biopesticides and other products against plant pathogen microorganisms. Therefore, the metabolism-related functions predicted in the endophytic bacteria community studied indicated that *S. dulcificum* harbors beneficial bacteria that could be exploited to improve host plant growth and synthesize secondary bioactive metabolites and antimicrobial compounds that inhibit the spread of soil disease, maintain host plant health and adaptation to various abiotic stresses.

## Conclusion

The diversity and structure of bacterial endophyte communities associated with the leaves and roots of *S. dulcificum* from its native environment were evaluated in this study. The endophytic bacteria belong to several phyla, with Actinomycetota, Pseudomonadota, and Chloroflexota being the most abundant in roots and Pseudomonadota and Actinomycetota in the leaves. Though the bacteria were diverse in each plant tissue, we observed a reduced leaf diversity compared to the root diversity. More importantly, the mineral content of the plant tissue, the rhizosphere soil chemical characteristics, accessions, phenotype, and habitat affected the endophytic bacterial communities that depend highly on the plant tissue type. Furthermore, and irrespective of the identified drivers, eight major OTUs, including OTU0001 (genus *Pseudomonas*), OTU0003 (*Sphingomonas*), OTU0004 (*Mycobacterium*), OTU0008 (unclassified Pseudomonadota), OTU0009 (unclassified Pseudomonadota), OTU0010 (unclassified Pseudomonadota), OTU0011 (unclassified Pseudomonadota), and OTU0083 (*Methyloversatilis*) were identified as a core bacterial community shared across the accessions studied. More importantly, functional metagenome prediction revealed that *S. dulcificum* shelters an endophytic bacterial community with potential roles in metabolism-related pathways and other essential functions. This endophytic bacterial community could, therefore, be harnessed for host plant growth improvement, secondary metabolites, and antimicrobial compounds synthesis for plant adaptation to biotic and abiotic stresses. Further characterization of OTUs identified for their growth-promoting traits could accelerate the valorization of this huge diversity in the development of sustainable and environmentally friendly approaches for increased productivity of *S. dulcificum*.

## Supporting information

S1 Table Distribution of the 29 *Synsepalum dulcificum* accessions sampled in Benin. (DOCX)

S2 Table Chemical characteristics of rhizosphere soils, roots and leaves of the 29 *Synsepalum dulcificum* accessions sampled in Benin. (XLSX)

S3 Table Alpha-diversity indices of leaf and root endophytic bacterial communities across *Synsepalum dulcificum* accessions. (DOCX)

S4 Table Relative abundance of the level 3 KEGG pathways based on gene profiles prediction of leaf and root endophytic bacterial communities of *Synsepalum dulcificum* using Tax4Fun 2. (DOCX)
