# Supplementary material for: Plant tissue type and mineral contents shape endophytic bacterial communities in the Sisrè berry plant [Synsepalum dulcificum (Schumach & Thonn.) Daniell] in Benin
Source: PLoS One. 2025 Jul 7;20(7):e0327715. doi: 10.1371/journal.pone.0327715 (PMC12233289; doi:10.1371/journal.pone.0327715)
Supplement: S1 Table — (DOCX) [file pone.0327715.s001.docx]

**S1 Table. Distribution of the 29 *Synsepalum dulcificum* accessions sampled in Benin.**

| **Accession** | **Habitat type** | **Phenotypic group** | **Latitude** | **Longitude** |
| --- | --- | --- | --- | --- |
| **1** | Home garden | 1 | N 6°55’30” | E 2°16’22.8” |
| **2** | Home garden | 3 | N 6°46’33.6” | E 2°06’14.4” |
| **3** | Farm | 2 | N 6°55’30” | E 1°57’10.8” |
| **4** | Home garden | 3 | N 6°33’41” | E 2°36’38.3” |
| **5** | Farm | 1 | N 6°55’02” | E 1°57’57.3” |
| **6** | Farm | 2 | N 6°50’20” | E 1°51’04.3” |
| **7** | Farm | 2 | N 7°03’06.3” | E 1°49’45.8” |
| **8** | Farm | 1 | N 6°59’15.6” | E 1°43’46.3” |
| **9** | Home garden | 2 | N 6°55’01.7” | E 1°57’28.2” |
| **10** | Home garden | 3 | N 6°32’46.6” | E 2°35’47.8” |
| **11** | Farm | 3 | N 6°52’27.4” | E 2°18’35.9” |
| **12** | Home garden | 1 | N 6°55’12” | E 2°16’19.2” |
| **13** | Home garden | 1 | N 6°55’46.3” | E 2°16’26.3” |
| **14** | Home garden | 3 | N 6°55’35.9” | E 2°16’06.02” |
| **15** | Farm | 2 | N 6°48’35.7” | E 2°17’54” |
| **16** | Home garden | 3 | N 7°13’04.8” | E 2°00’20.3” |
| **17** | Home garden | 2 | N 6°55’44.4” | E 2°16’22.8” |
| **18** | Home garden | 1 | N 7°9’23.2” | E 2°03’32” |
| **19** | Home garden | 1 | N 6°54’53.8” | E 1°50’45.6” |
| **20** | Home garden | 3 | N 7°06’59” | E 1°59’56” |
| **21** | Home garden | 2 | N 6°55’01.2” | E 2°16’22.8” |
| **22** | Home garden | 1 | N 6°38’26.7” | E 2°33’16.3” |
| **23** | Home garden | 3 | N 6°33’40.8” | E 2°36’38.3” |
| **24** | Farm | 1 | N 6°40’32.7” | E 2°16’10.3” |
| **25** | Home garden | 2 | N 7°13’29.5” | E 2°20’09.6” |
| **26** | Home garden | 1 | N 6°21’49.6” | E 1°54’01.4” |
| **27** | Home garden | 1 | N 6°26’08.1” | E 1°50’29” |
| **28** | Home garden | 2 | N 6°45’54” | E 2°06’21.6” |
| **29** | Home garden | 3 | N 6°58’57.3” | E 2°38’42.7” |
