# Supplementary material for: Plant tissue type and mineral contents shape endophytic bacterial communities in the Sisrè berry plant [Synsepalum dulcificum (Schumach & Thonn.) Daniell] in Benin
Source: PLoS One. 2025 Jul 7;20(7):e0327715. doi: 10.1371/journal.pone.0327715 (PMC12233289; doi:10.1371/journal.pone.0327715)
Supplement: S3 Table — (DOCX) [file pone.0327715.s003.docx]

**S1 Table. Alpha-diversity indices of leaf and root endophytic bacterial communities across *Synsepalum dulcificum* accessions.**

| **Plant tissue** | **Accession** | **Observed OTUs** | **Chao1 estimator** | **Shannon-Wiener Index H’** | **Shannon Index Evenness E** | **Inverse Simpson Index1/D** | **Good’s coverage** |
| --- | --- | --- | --- | --- | --- | --- | --- |
| **Leaf** | 1 | 57.33 ± 2.40 a | 92.33 ± 1.13 a | 1.10 ± 0.04 a | 0.27 ± 0.01 a | 1.74 ± 0.05 a | 0.99 ± 0.00 a |
|  | 2 | 47.00 ± 5.13 a | 84.52 ± 22.45 a | 0.99 ± 0.04 a | 0.26 ± 0.00 a | 1.61 ± 0.03 a | 0.99 ± 0.00 a |
|  | 3 | 71.00 ± 33.00 a | 148.38 ± 61.48 a | 1.19 ± 0.28 a | 0.29 ± 0.04 a | 1.84 ± 0.25 a | 0.99 ± 0.01 a |
|  | 4 | 36.67 ± 2.19 a | 60.54 ± 1.31 a | 0.95 ± 0.04 a | 0.26 ± 0.01 a | 1.60 ± 0.05 a | 1.00 ± 0.00 a |
|  | 5 | 58.00 ± 4.62 a | 94.42 ± 7.78 a | 1.15 ± 0.10 a | 0.28 ± 0.02 a | 1.87 ± 0.13 a | 0.99 ± 0.00 a |
|  | 6 | 52.67 ± 5.21 a | 92.85 ± 15.84 a | 1.03 ± 0.03 a | 0.26 ± 0.00 a | 1.65 ± 0.03 a | 0.99 ± 0.00 a |
|  | 7 | 53.67 ± 6.74 a | 89.45 ± 7.94 a | 1.07 ± 0.08 a | 0.27 ± 0.01 a | 1.68 ± 0.06 a | 0.99 ± 0.00 a |
|  | 8 | 59.00 ± 10.97 a | 87.95 ± 16.35 a | 1.19 ± 0.12 a | 0.29 ± 0.02 a | 1.82 ± 0.09 a | 0.99 ± 0.00 a |
|  | 9 | 47.00 ± 3.61 a | 106.38 ± 27.81 a | 0.98 ± 0.02 a | 0.26 ± 0.00 a | 1.62 ± 0.01 a | 0.99 ± 0.00 a |
|  | 10 | 67.33 ± 14.75 a | 116.59 ± 22.46 a | 1.13 ± 0.11 a | 0.27 ± 0.01 a | 1.66 ± 0.06 a | 0.99 ± 0.00 a |
|  | 11 | 47.33 ± 1.86 a | 77.64 ± 1.88 a | 1.00 ± 0.03 a | 0.26 ± 0.00 a | 1.62 ± 0.01 a | 0.99 ± 0.00 a |
|  | 12 | 34.00 ± 2.08 a | 53.73 ± 7.75 a | 0.96 ± 0.06 a | 0.27 ± 0.01 a | 1.64 ± 0.07 a | 1.00 ± 0.00 a |
|  | 13 | 43.67 ± 9.70 a | 55.98 ± 9.73 a | 1.00 ± 0.10 a | 0.27 ± 0.01 a | 1.61 ± 0.06 a | 1.00 ± 0.00 a |
|  | 14 | 38.67 ± 1.86 a | 56.18 ± 7.50 a | 1.01 ± 0.10 a | 0.28 ± 0.03 a | 1.64 ± 0.10 a | 1.00 ± 0.00 a |
|  | 15 | 43.67 ± 4.37 a | 71.14 ± 14.86 a | 1.14 ± 0.08 a | 0.30 ± 0.03 a | 1.78 ± 0.11 a | 0.99 ± 0.00 a |
|  | 16 | 65.33 ± 13.93 a | 127.95 ± 9.13 a | 1.12 ± 0.08 a | 0.27 ± 0.01 a | 1.79 ± 0.09 a | 0.99 ± 0.00 a |
|  | 17 | 45.33 ± 6.84 a | 78.21 ± 15.53 a | 1.03 ± 0.12 a | 0.27 ± 0.02 a | 1.70 ± 0.14 a | 0.99 ± 0.00 a |
|  | 18 | 50.67 ± 6.89 a | 80.75 ± 11.96 a | 1.01 ± 0.03 a | 0.26 ± 0.00 a | 1.59 ± 0.02 a | 0.99 ± 0.00 a |
|  | 19 | 43.67 ± 9.21 a | 93.89 ± 19.11 a | 0.87 ± 0.09 a | 0.23 ± 0.01 a | 1.52 ± 0.05 a | 0.99 ± 0.00 a |
|  | 20 | 67.67 ± 22.75 a | 104.65 ± 32.59 a | 1.03 ± 0.15 a | 0.25 ± 0.02 a | 1.56 ± 0.08 a | 0.99 ± 0.00 a |
|  | 21 | 64.33 ± 10.17 a | 84.70 ± 10.61 a | 1.07 ± 0.09 a | 0.26 ± 0.01 a | 1.60 ± 0.06 a | 0.99 ± 0.00 a |
|  | 22 | 50.00 ± 8.33 a | 84.58 ± 15.57 a | 0.91 ± 0.09 a | 0.23 ± 0.01 a | 1.52 ± 0.07 a | 0.99 ± 0.00 a |
|  | 23 | 54.00 ± 3.06 a | 95.43 ± 12.87 a | 1.25 ± 0.08 a | 0.31 ± 0.02 a | 1.98 ± 0.28 a | 0.99 ± 0.00 a |
|  | 24 | 87.67 ± 4.41 a | 135.10 ± 3.24 a | 1.36 ± 0.09 a | 0.30 ± 0.02 a | 1.84 ± 0.10 a | 0.99 ± 0.00 a |
|  | 25 | 40.00 ± 2.31 a | 64.03 ± 10.66 a | 0.91 ± 0.06 a | 0.25 ± 0.02 a | 1.56 ± 0.09 a | 0.99 ± 0.00 a |
|  | 26 | 65.00 ± 17.69 a | 98.52 ± 26.84 a | 1.22 ± 0.15 a | 0.29 ± 0.02 a | 1.75 ± 0.08 a | 0.99 ± 0.00 a |
|  | 27 | 48.00 ± 10.69 a | 74.31 ± 14.66 a | 1.03 ± 0.19 a | 0.27 ± 0.03 a | 1.69 ± 0.17 a | 0.99 ± 0.00 a |
|  | 28 | 39.67 ± 1.45 a | 64.75 ± 8.42 a | 0.87 ± 0.03 a | 0.24 ± 0.01 a | 1.55 ± 0.04 a | 0.99 ± 0.00 a |
|  | 29 | 46.67 ± 4.81 a | 78.03 ± 20.06 a | 1.00 ± 0.01 a | 0.26 ± 0.00 a | 1.62 ± 0.01 a | 0.99 ± 0.00 a |
| **Root** | 1 | 302.67 ± 17.89 ab | 455.94 ± 45.16 ab | 3.72 ± 0.27 ab | 0.65 ± 0.04 a | 14.59 ± 5.65 a | 0.96 ± 0.00 bcd |
|  | 2 | 280.00 ± 14.43 ab | 428.03 ± 5.19 ab | 3.39 ± 0.35 ab | 0.60 ± 0.06 a | 11.30 ± 3.63 a | 0.97 ± 0.00 bcd |
|  | 3 | 257.33 ± 9.49 abc | 425.60 ± 11.53 abc | 3.46 ± 0.13 ab | 0.62 ± 0.02 a | 10.81 ± 2.91 a | 0.97 ± 0.00 abcd |
|  | 4 | 274.33 ± 26.59 ab | 366.30 ± 42.27 abc | 3.97 ± 0.07 a | 0.71 ± 0.01 a | 19.08 ± 2.06 a | 0.97 ± 0.00 abcd |
|  | 5 | 196.67 ± 15.34 abc | 336.41 ± 41.16 abc | 2.69 ± 0.22 ab | 0.51 ± 0.04 a | 4.94 ± 1.10 a | 0.97 ± 0.00 abcd |
|  | 6 | 219.67 ± 25.05 abc | 349.99 ± 22.50 abc | 3.32 ± 0.18 ab | 0.62 ± 0.02 a | 11.10 ± 2.28 a | 0.97 ± 0.00 abcd |
|  | 7 | 290.00 ± 21.22 ab | 444.97 ± 51.27 ab | 3.63 ± 0.22 ab | 0.64 ± 0.03 a | 13.20 ± 3.09 a | 0.96 ± 0.00 bcd |
|  | 8 | 189.33 ± 20.85 abc | 293.81 ± 29.79 abc | 3.06 ± 0.31 ab | 0.58 ± 0.05 a | 9.01 ± 3.91 a | 0.98 ± 0.00 abcd |
|  | 9 | 289.00 ± 13.23 ab | 466.35 ± 23.78 ab | 3.56 ± 0.09 ab | 0.63 ± 0.01 a | 11.51 ± 1.01 a | 0.96 ± 0.00 cd |
|  | 10 | 212.67 ± 28.29 abc | 299.32 ± 32.56 abc | 3.13 ± 0.24 ab | 0.59 ± 0.05 a | 8.28 ± 3.26 a | 0.98 ± 0.00 abcd |
|  | 11 | 261.67 ± 19.81 abc | 409.03 ± 35.32 abc | 3.37 ± 0.09 ab | 0.61 ± 0.01 a | 9.72 ± 1.51 a | 0.97 ± 0.00 abcd |
|  | 12 | 305.33 ± 18.49 ab | 459.41 ± 35.13 ab | 3.8 ± 0.12 ab | 0.66 ± 0.02 a | 15.87 ± 2.91 a | 0.96 ± 0.00 bcd |
|  | 13 | 284.33 ± 17.68 ab | 439.23 ± 39.90 ab | 3.67 ± 0.25 ab | 0.65 ± 0.04 a | 13.10 ± 4.79 a | 0.97 ± 0.00 bcd |
|  | 14 | 289.67 ± 27.29 ab | 441.28 ± 60.28 ab | 3.69 ± 0.28 ab | 0.65 ± 0.04 a | 13.75 ± 4.19 a | 0.97 ± 0.00 bcd |
|  | 15 | 275.00 ± 28.29 ab | 423.82 ± 34.93 abc | 3.65 ± 0.18 ab | 0.65 ± 0.02 a | 14.69 ± 3.37 a | 0.97 ± 0.00 bcd |
|  | 16 | 288.33 ± 10.68 ab | 457.98 ± 38.89 ab | 3.53 ± 0.27 ab | 0.62 ± 0.05 a | 14.00 ± 3.94 a | 0.96 ± 0.00 bcd |
|  | 17 | 248.00 ± 18.56 abc | 413.78 ± 12.16 abc | 3.4 ± 0.24 ab | 0.62 ± 0.04 a | 11.24 ± 2.85 a | 0.97 ± 0.00 abcd |
|  | 18 | 131.67 ± 32.10 c | 190.57 ± 34.84 c | 2.36 ± 0.50 b | 0.48 ± 0.08 a | 5.53 ± 2.13 a | 0.99 ± 0.00 a |
|  | 19 | 230.33 ± 22.39 abc | 326.11 ± 46.51 abc | 3.29 ± 0.16 ab | 0.61 ± 0.02 a | 8.03 ± 1.08 a | 0.98 ± 0.00 abcd |
|  | 20 | 300.67 ± 25.01 ab | 486.72 ± 30.32 ab | 3.76 ± 0.17 ab | 0.66 ± 0.02 a | 16.86 ± 2.67 a | 0.96 ± 0.00 d |
|  | 21 | 181.00 ± 50.24 bc | 258.32 ± 68.93 bc | 2.37 ± 0.69 b | 0.45 ± 0.11 a | 4.74 ± 2.67 a | 0.98 ± 0.01 abc |
|  | 22 | 224.33 ± 23.79 abc | 307.65 ± 25.94 abc | 3.61 ± 0.25 ab | 0.67 ± 0.03 a | 16.34 ± 3.59 a | 0.98 ± 0.00 abcd |
|  | 23 | 192.67 ± 5.90 abc | 264.75 ± 9.63 abc | 3.23 ± 0.14 ab | 0.61 ± 0.03 a | 9.50 ± 3.32 a | 0.98 ± 0.00 ab |
|  | 24 | 247.00 ± 29.94 abc | 392.35 ± 56.37 abc | 3.57 ± 0.27 ab | 0.65 ± 0.03 a | 16.19 ± 3.76 a | 0.97 ± 0.00 abcd |
|  | 25 | 253.33 ± 27.49 abc | 383.84 ± 97.52 abc | 3.43 ± 0.23 ab | 0.62 ± 0.03 a | 10.11 ± 2.17 a | 0.97 ± 0.01 abcd |
|  | 26 | 193.00 ± 12.00 abc | 309.37 ± 16.03 abc | 2.84 ± 0.17 ab | 0.54 ± 0.03 a | 6.53 ± 0.89 a | 0.98 ± 0.00 abcd |
|  | 27 | 191.00 ± 28.31 abc | 307.22 ± 49.32 abc | 2.83 ± 0.52 ab | 0.54 ± 0.08 a | 8.29 ± 4.56 a | 0.98 ± 0.00 abcd |
|  | 28 | 225.00 ± 39.04 abc | 357.09 ± 74.29 abc | 2.89 ± 0.46 ab | 0.53 ± 0.07 a | 6.44 ± 2.57 a | 0.97 ± 0.01 abcd |
|  | 29 | 320.67 ± 12.41 a | 498.05 ± 5.51 a | 3.88 ± 0.24 ab | 0.67 ± 0.04 a | 18.29 ± 6.98 a | 0.96 ± 0.00 d |

Statistics were performed using an OTU threshold of ≥ 97% sequence similarity on randomly sub-sampled data at the lower sample size (3,547 reads). For each factor, values (mean ± standard error) within a column followed by the different letters were significantly different according to Tukey’s HSD post-hoc test at p < 0.05.
