# Supplementary material for: Plant tissue type and mineral contents shape endophytic bacterial communities in the Sisrè berry plant [Synsepalum dulcificum (Schumach & Thonn.) Daniell] in Benin
Source: PLoS One. 2025 Jul 7;20(7):e0327715. doi: 10.1371/journal.pone.0327715 (PMC12233289; doi:10.1371/journal.pone.0327715)
Supplement: S4 Table — (DOCX) [file pone.0327715.s004.docx]

**S2 Table. Relative abundance of the level 3 KEGG pathways based on gene profiles prediction of leaf and root endophytic bacterial communities of *Synsepalum dulcificum* using Tax4Fun 2.**

| **Level 3 KEGG pathway** | **Relative abundance in the leaf (%)** | **Relative abundance in the root (%)** | **Wilcoxon rank test (p < 0.05)** |
| --- | --- | --- | --- |
| **Metabolism** | 70.39 | 80.10 | p<0.001 |
| **Environmental Information Processing** | 13.22 | 7.91 | p<0.001 |
| **Cellular Processes** | 8.44 | 5.25 | p<0.001 |
| **Genetic Information Processing** | 2.81 | 2.42 | p<0.001 |
| **Organismal Systems** | 1.35 | 1.95 | p<0.001 |
| **Human diseases** | 3.80 | 2.37 | p<0.001 |
